# Decoding Specificity of Cyanobacterial MysDs in Mycosporine-like Amino Acid Biosynthesis through Heterologous Expression in *Saccharomyces cerevisiae*

**DOI:** 10.1021/acsomega.5c01035

**Published:** 2025-03-28

**Authors:** Xiaoyou Zheng, Peifeng Xie, Andrew Chen Cai, Yuze Jiang, Sirui Huang, Xiaochong Ma, Honghao Su, Boxiang Wang

**Affiliations:** † Churchill College, University of Cambridge, Storey’s Way, Cambridge CB3 0DS, U.K.; ‡ LINK SPIDER Co., Ltd., 11 Langshan Rd, Nanshan District, Shenzhen 518000, China; § Thurgood Marshall College, University of California, 9500 Gilman Dr., La Jolla, San Diego, California 92093, United States; ∥ Earlham Institute, Norwich Research Park, Norwich NR4 7UZ, U.K.

## Abstract

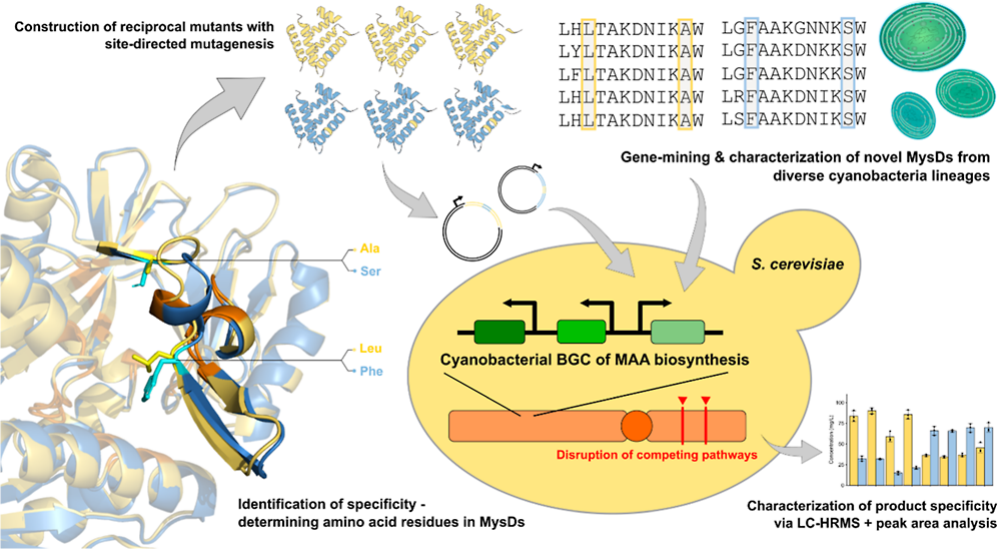

Mycosporine-like amino acids (MAAs) are potent natural UV-protectants, but their industrial production is hindered by efficiency and sustainability issues in large-scale extraction of native hosts. Heterologous biosynthesis in chassis organisms provides a promising alternative route, although the substrate promiscuity of the ATP-grasp ligase MysD complicates the biosynthesis of specific MAAs. In this study, we developed a *Saccharomyces cerevisiae* strain with enhanced capacity of producing mycosporine-glycine (MG). This strain serves as an efficient MysD expression platform that converts MG into shinorine and porphyra-334. Through structural modeling, site-directed mutagenesis, and mutant characterization, we identified two residues on the omega-loop of MysD involved in determining product specificity. We further characterized the product specificity of 20 MysDs from diverse cyanobacterial lineages and confirmed the residue pattern-product specificity correlation. Our findings provide guidance for screening, selecting, and designing novel MysDs for industrial-scale MAA production through heterologous expression.

## Introduction

Prolonged exposure to ultraviolet radiation (UVR) from the sun is associated with increased risk of erythema, oxidative injury, photoaging, and skin cancer, creating a need of effective sunscreens.^1^ Mycosporine-like amino acids (MAAs), photoprotectants capable of absorbing both UVA (315–400 nm) and UVB (280–315 nm), are found in diverse UV-adapted organisms, including several lineages of actinobacteria, cyanobacteria, and diatoms.^2−5^ Compared to conventional sunscreens with zinc oxide and oxybenzone content, MAAs demonstrate higher biocompatibility and less environmental impact, which make them promising natural sunscreens for industrial production.^6,7^ Traditional method of MAA production, which involves large-scale harvesting and extraction of marine algae, suffers from low overall yield and product purity and raises the issue of profitability and sustainability.^8^ As an alternative means, heterologous production in microorganisms including *Escherichia coli* and *Saccharomyces cerevisiae* through pathway engineering demonstrates potential for more efficient and specific production of valuable natural products, including MAAs.^9,10^ However, engineering MAA production in heterologous hosts requires knowledge of the MAA biosynthesis pathway.

**Figure 1:**
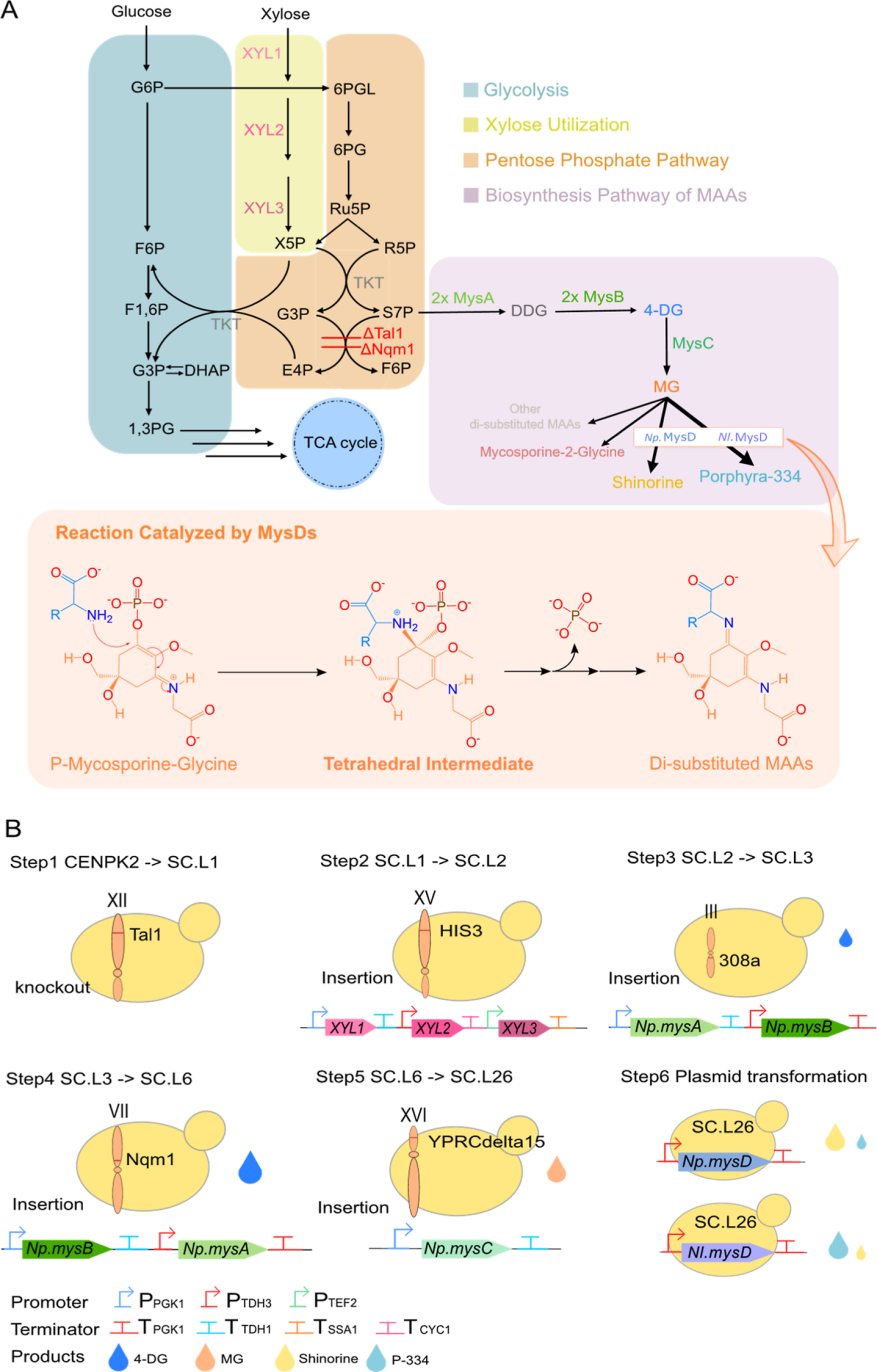
(A) Engineered metabolic pathway for the biosynthesis of disubstituted MAAs. The knockout of the transaldolase gene *TAL1* and its paralog *NQM1* (Red) limits the conversion of S7P into the unwanted F6P. The introduction of the xylose-utilizing pathway *xyl1*, *2*, and 3 (Magenta) enhances S7P production by directing xylose into PPP. The double knock-in of *mysA* and *mysB* and the introduction of *mysC* (Green) achieve the biosynthesis of MG. *mysDs* including *Np.mysD* and *Nl.mysD* (Blue and Purple) direct the synthesis of disubstituted MAAs including shinorine and porphyra-334 (Yellow and Blue). The reaction mechanism from MG to disubstituted MAAs is shown. The tetrahedral intermediate (middle) was used for molecular docking. Abbreviations of metabolites are provided in the glossary of Supporting Information. (B) Stepwise yeast engineering for MAA production. The orange bubbles represent individual chromosomes, whereas sites of knockout and insertion are labeled as red dashes. The knock-in of the second copy of *mysA* and *mysB* and the deletion of the *NQM1* gene were simultaneously accomplished in step4.

Biosynthetic gene clusters (BGCs) of MAA have been discovered in several cyanobacteria strains, and most of them contain three conserved genes, including *mysA* which encodes a DDG synthase (DDGS), *mysB* which encodes a SAM-dependent *O*-methyltransferase (*O*-MT), and *mysC* which encodes an ATP-grasp enzyme.^11^ MysA and MysB catalyze the stepwise conversion of sedoheptulose 7-phosphate (S7P) from the pentose phosphate pathway (PPP) into 4-deoxygadusol (4-DG), while MysC subsequently adds glycine to 4-DG to form mycosporine-glycine (MG) (Figure 1A). Several BGCs also include *mysD*, which encodes a homologue of d-alanine-d-alanine ligase.^11,12^ MysD catalyzes the conjugation of an amino acid to MG, which gives rise to different types of disubstituted MAAs. Conjugation of serine, threonine, and glycine with MG gives rise to shinorine, porphyra-334, and mycosporine 2-glycine, respectively (Figure 1A).^13,14^

According to previous analysis on the mode-of-action of d-alanine-d-alanine ligases and predicted catalytic mechanism of MysDs,^12,15,16^ the reaction from MG to disubstituted MAAs begins with the phosphorylation of the C1 position of MG, followed by a nucleophilic attack from the amine moiety of the amino acid substrate on the same position (C1). These actions result in the formation of a tetrahedral phosphate-containing intermediate (Figures 1A, S5). The reaction is completed by phosphate group dissociation and the release of MAA product and ADP.

All characterized MysDs show product promiscuity and produce more than one type of disubstituted MAAs, mostly a mixture of shinorine and porphyra-334.^14^ However, shinorine-to-porphyra-334 ratio varies among different MysDs.^12−14,17,18^ Nonetheless, besides Kim et al., 2023, which correlated product specificity with an omega loop, few studies have explored the molecular basis of the difference in product specificity.^14^ Hence, in this study, we explored the molecular basis of MysD product specificity to guide its engineering to enhance the efficiency and specificity of shinorine and porphyra-334 production. We developed an MG-producing yeast strain for wild-type and mutant MysD characterization and found that product specificity boils down to two residues. We subsequently validated their importance by characterizing uncharacterized MysDs with different compositions of these residues.

## Results and Discussion

### Engineering Yeast Strains for MG Production

MG is the common precursor of disubstituted MAAs shinorine and porphyra-334 (Figure 1A). Therefore, a *S. cerevisiae* strain efficient at producing MG needs to be developed as a shinorine and porphyra-334 production platform. Based on Park et al., 2019’s strategy,^18^ we sequentially knocked out the transaldolase gene *TAL1*, which directs S7P flux into PPP, and engineered a xylose-utilization pathway by inserting three *Scheffersomyces stipitis* xylose-utilizing genes (*XYL1*, *XYL2*, and *XYL3*) in *HIS3* locus in *S. cerevisiae* strain CEN-PK to enhance S7P production, resulting in SC.L2 (Figure 1A,B).

**Figure 2:**
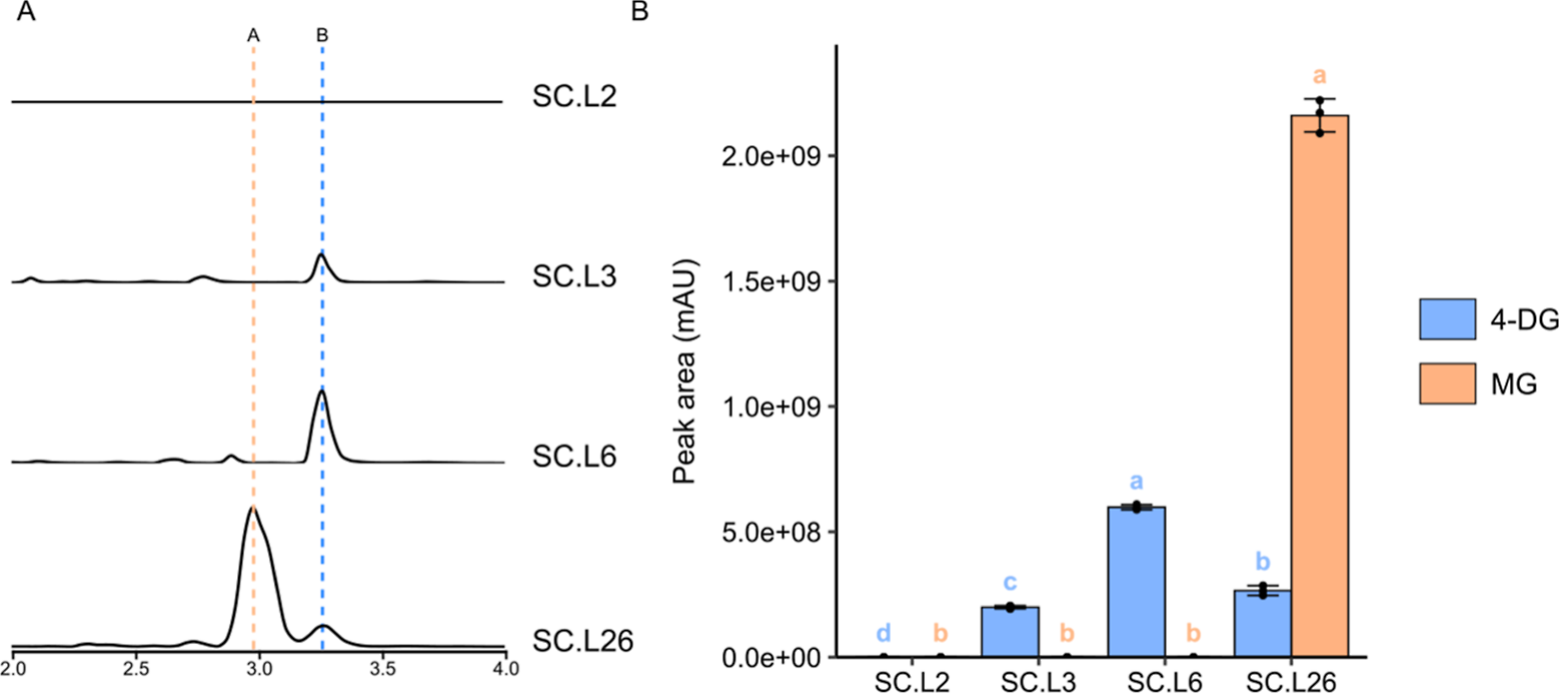
Engineering *S. cerevisiae* for MG production. (A) Extracted ion chromatograms (EICs) of LC-HRMS analysis of ions with *m*/*z* = 188.08–189.08 and 245.10–246.10 in SC.L2, SC.L3, SC.L6, and SC.L26 samples. Peak B’s retention time is 3.24 min and peak A’s retention time is 2.93 min, and their identities were confirmed as 4-DG and MG through MS analysis. The height of each chromatogram is 2.5 × 10^8^ arbitrary unit. (B) 4-DG and MG peak area comparison among the yeast strains. Heights of the bars represent their means, and the error bars represent their standard deviations (*n* = 3 biological replicates). One-way ANOVA was performed to compare the amount of 4-DG and MG separately among different samples, and two samples that do not share the same letter are significantly different from each other (*p* < 0.05).

To convert S7P into 4-DG, we sequentially made strains SC.L3 and SC.L6 by inserting either just one copy of *Np.mysA* and *Np.mysB* from *Nostoc punctiforme* into 308 locus or another copy of them into *NQM1* locus, a paralog of *TAL1* (Figure 1A,B). To confirm their 4-DG production capability, both strains were cultured and extracted for LC-HRMS analysis (Figure 2A). The 4-DG peak was observed in the chromatograms of SC.L3 and SC.L6 but not SC.L2. Through peak area comparison, we observed a significant increase in 4-DG production in SC.L6 compared to that in SC.L3 (*p* < 0.05, Tukey’s HSD) with a 3.00-fold difference (Figure 2B). SC.L6 was therefore used for engineering further steps.

To complete the MG production pathway, we inserted *Np.mysC* into locus *YPRCdelta15* of SC.L6 to form strain SC.L26 (Figure 1A,B). SC.L26 was cultured and extracted for LC-HRMS analysis, and both 4-DG and MG peaks were observed in the chromatogram (Figure 2A).

### Identification of Two Specificity-Determining Residues on the Omega-Loop of MysD

**Figure 3:**
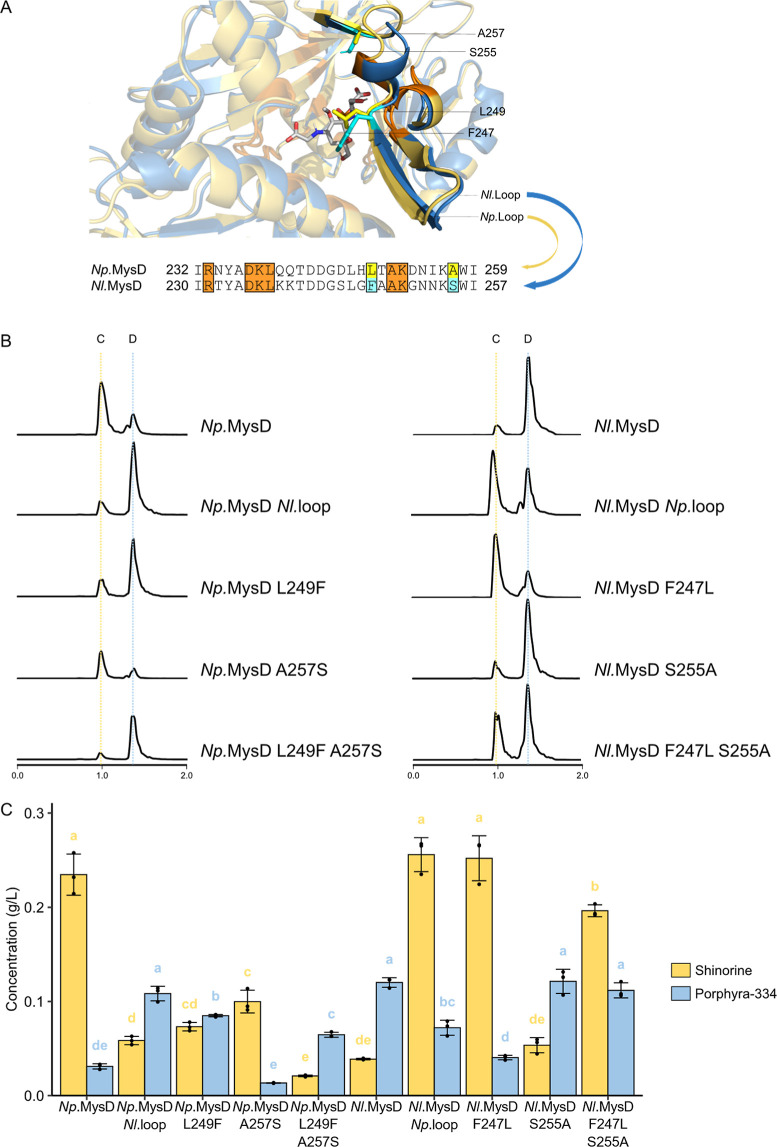
(A) Predicted structural models of *Np.*MysD (blue) and *Nl.*MysD (yellow) with a reaction intermediate (M-Gly-P-Ser) docked to active sites and an alignment of their omega-loop sequences. The identical sequences within 5 Å from the intermediate are colored orange. The two critical amino acid residues are highlighted with yellow (*Np.*MysD) or cyan (*Nl.*MysD). (B) EICs of LC-HRMS analysis of ions with *m*/*z* = 332.50–333.50 and 346.50–347.50 in SC.L26 expressing native and mutant *Np.*MysD and *Nl.*MysD. Peak C’s retention time is 1.00 min and peak D’s retention time is 1.38 min, and their identities were confirmed as shinorine and porphyra-334 through MS analysis. The height of each chromatogram is 1.2 × 10^9^ arbitrary unit. (C) Amount of shinorine and porphyra-334 produced by native and mutant *Np.*MysD and *Nl.*MysD. Heights of the bars represent their means, and the error bars represent their standard deviations (*n* = 3 biological replicates). One-way ANOVA was performed to compare the amount of shinorine and porphyra-334 separately among different samples, and two samples that do not share the same letter are significantly different from each other (*p* < 0.05).

Development of MG-producing SC.L26 enables the characterization of MysD product specificity through heterologous expression. To look for product specificity determinants, we selected MysDs from *N. punctiforme* PCC 73102 (*Np.*MysD) and *Nostoc linckia* NIES-25 (*Nl.*MysD) as exemplars of shinorine-preferred producer and porphyra-334-preferred producer, respectively (Figure 1A) and acquired their structural models through ColabFold. Structural models of both *Np.*MysD and *Nl.*MysD showed high structural similarity with *E. coli*d-alanine-d-alanine ligase’s crystal structure^16^ (PDB ID: 2DLN, RMSD = 1.901 and 1.794 respectively). Hence, we predicted their active site pockets based on 2DLN and docked the tetrahedral intermediate (MG-P-Ser) there (Figures 3A, S1). We hypothesized that residues less than 8 Å away from the docked intermediates that differ between *Np.*MysD and *Nl.*MysD are involved in substrate recognition and hence product specificity determination.

All active site residues that differ between *Np.*MysD and *Nl.*MysD cluster in a 26-amino acid long segment located on the omega-loop discovered by Kim et al., 2023 (Figures 3A, S2).^14^ We performed loop substitution on both *Np.*MysD and *Nl.*MysD and expressed wild-type and loop-substituted *Np.*MysDs and *Nl.*MysDs in SC.L26. By performing LC-HRMS, we found that *Np.*MysD-*Nl.*Loop produces predominantly porphyra-334, while *Nl.*MysD-*Np.*Loop produces predominantly shinorine, both of which display a reverse in dominant product compared to their corresponding wild-types (Figure 3B), which corroborates on the previous study.^14^ Further metabolite content analysis shows a significant reduction in dominant product and a significant increase in nondominant product in the loop-substitution mutants (Figure 3B). Nevertheless, we observed a reduction in total activity in *Np.*MysD-*Nl.*Loop, while an enhancement in total activity occurred in *Nl.*MysD-*Np.*Loop (Figure S3).

We then narrowed the scope to residues less than 5 Å away from the intermediate and found only two residues that differ between *Np.*MysD and *Nl.*MysD (Figure 3A,B). We hypothesized that the difference in product specificity between *Np.*MysD and *Nl.*MysD arises from the differential interactions of these two residues with the reaction intermediate. To validate the role of these two residues, we created single and double *Np.*MysD and *Nl.*MysD reciprocal mutants and expressed them in SC.L26.

According to EIC, for both *Np.*MysD and *Nl.*MysD, a switch in dominant product was observed in L249F/F247L single mutants and L249F-A257S double mutants, with *Np.*MysD mutants producing predominantly porphyra-334 (peak C) and *Nl.*MysD mutants producing predominantly shinorine (peak D) (Figure 3B). However, based on the EIC, the switch was not observed in *Np.*MysD-A257S, *Nl.*MysD-S255A, and *Nl.*MysD-F247L-S255A. Nevertheless, in this LC-HRMS run, the machine is 2.7 times more sensitive to porphyra-334 than shinorine. In specific, the peak area of the 0.1 mg/mL porphyra-334 standard is 2.7 times greater than that of the 0.1 mg/mL shinorine standard. Hence, the peak sizes from EIC might not accurately reflect the actual content. Therefore, we calculated the concentrations of shinorine and porphyra-334 in all samples by comparing peak areas with the peak areas of standards (Figure 3C).

In metabolite content analysis, we found a significant reduction in shinorine production in all *Np.*MysD mutants and a significant increase in porphyra-334 production in all mutants except *Np.*MysD-A257S (Figure 3C), suggesting that the two residues picked are involved in determining the product specificity in *Np.*MysD. The *Np.*MysD-L249F-A257S double mutant shows a higher porphyra-334-to-shinorine ratio than both single mutants, suggesting potential concerted action between the two residues. However, a significant decline in the overall activity was observed in all *Np.*MysD mutants (Figure S3).

For *Nl.*MysD, a significant increase in shinorine production was observed in both *Nl.*MysD-F247L and *Nl.*MysD-F247L-S255A mutants, while a significant reduction in porphyra-334 production was observed only in the *Nl.*MysD-F247L mutant (Figure 3C). No significant difference in shinorine or porphyra-334 production was observed in the *Nl.*MysD-S255A mutant compared to wild-type. The *Nl.*MysD-F247L single mutant shows a higher shinorine-to-porphyra-334 ratio than the other two mutants, suggesting that the S255A mutation might reduce the effect of the F247L mutation. In addition, we observed a significant boost in activity in both *Nl.*MysD-F247L and *Nl.*MysD-F247L-S255A mutants compared to wild-type (Figure S3).

The mutagenesis results narrowed the product specificity determinant of MysD down to the identities of two residues on the omega loop. Based on metabolite content produced by wild-type and mutants *Np.*MysD and *Nl.*MysD, we showed a pattern in which MysD preferentially produces shinorine if the two residues are L–A or L–S, while MysD preferentially produces porphyra-334 if the two residues are F–S or F–A. The pattern of these two residues could provide guidance in screening for more efficient and specific shinorine or porphyra-334 producers.

### Characterization of Product Specificity of Various Cyanobacterial MysD Orthologs Selected Based on Specificity-Determining Residues

**Figure 4:**
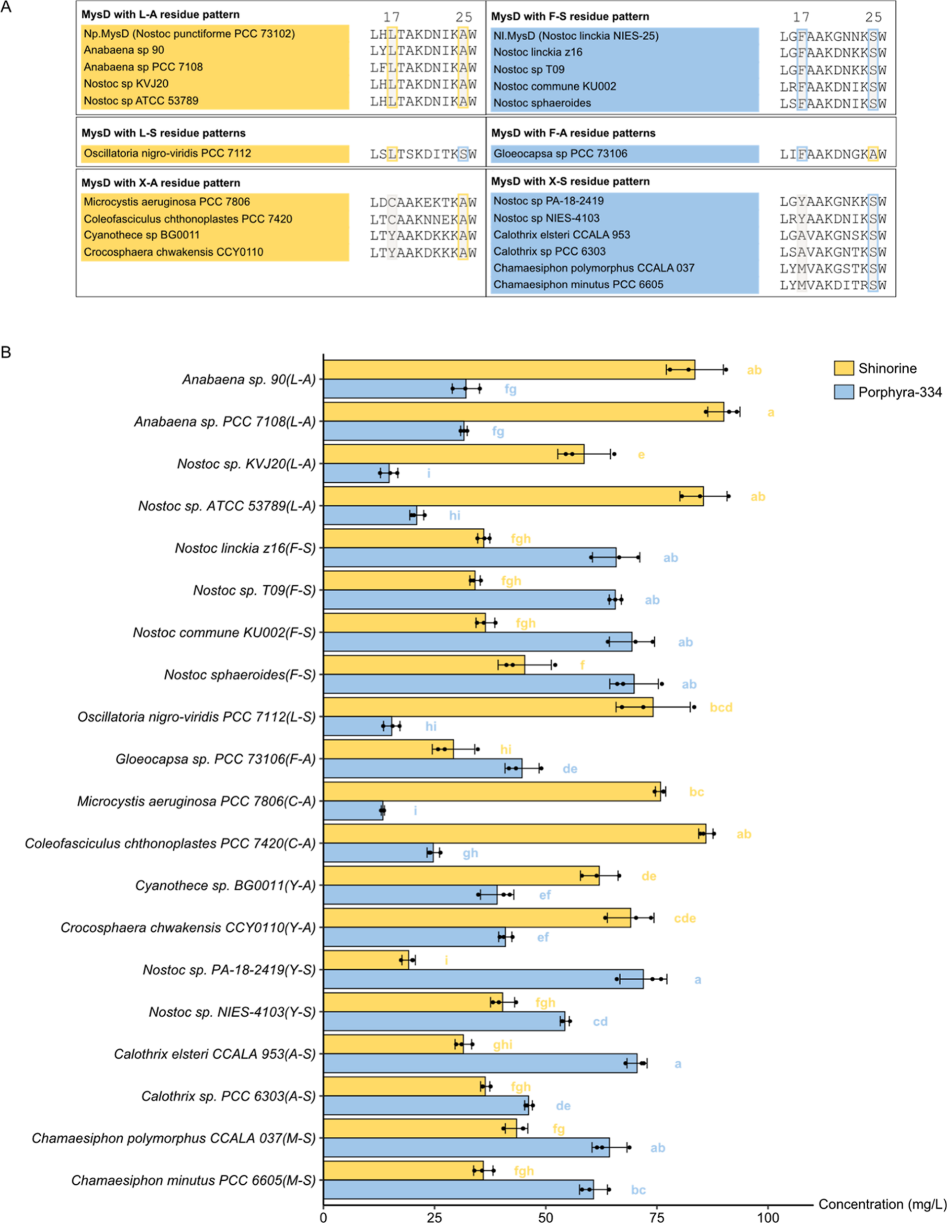
(A) Classification of MysD sequences characterized in this study based on the pattern of two key residues. Sequences hypothesized to produce more shinorine are labeled in yellow, while those hypothesized to produce more porphyra-334 are labeled in blue. Two key residues are highlighted by rectangles with gray background color. (B) Shinorine and porphyra-334 production from LC-HRMS peak area analysis by yeast strains expressing selected MysDs. Lengths of the bars represent their means, and the error bars represent their standard deviations (*n* = 3 biological replicates). One-way ANOVA was performed to compare the amount of shinorine and porphyra-334 separately among different samples, and two samples that do not share the same letter are significantly different from each other (*p* < 0.05).

To validate the role of these two residues in determining product specificity, we set out to characterize other MysD homologues with varied residue pattern. We interrogated OrthoDB and NCBI databases with BLASTp using *Np.*MysD and *Nl.*MysD as query sequences, performed multiple sequence alignment of the hits, and chose 20 more sequences to characterize (Figure 4A). We acquired their coding sequences through gene synthesis and expressed them in SC.L26. LC-HRMS followed by peak area extraction and metabolite content quantification were applied to evaluate the amount of shinorine and porphyra-334 produced by each MysD.

We first hypothesized that all MysDs with an L–A pattern (same as wild-type *Np.*MysD) preferentially produce shinorine, while all MysDs with an F–S pattern (same as wild-type *Nl.*MysD) preferentially produce porphyra-334. Therefore, we chose four MysDs with the L–A pattern (*Anabaena* sp. 90, *Anabaena* sp. PCC 7108, *Nostoc* sp. KVJ20, *Nostoc* sp. ATCC 53789) and four MysDs with F–S patterns (*N. linckia* z16, *Nostoc* sp. T09, *Nostoc commune* KU002, *Nostoc sphaeroides*) for characterization. Consistent with our hypothesis, all MysDs selected with the L–A pattern produce more shinorine, while all MysDs selected with the F–S pattern produce more porphyra-334 when expressed in SC.L26 (Figures 4B, S9–S16).

In the mutagenesis experiment, we found that the *Nl.*MysD-F247L mutant which shows the L–S pattern preferentially produces shinorine, while the *Np.*MysD-L249F mutant which shows the F–A pattern preferentially produces porphyra-334. To test if the pattern-product specificity relationship also holds true for natural MysDs, we expressed one MysD with an L–S pattern (*Oscillatoria nigro-viridis* PCC 7112) and one MysD with an F–A pattern (*Gloeocapsa* sp. PCC 73106) in SC.L26, and their product specificities were consistent with our expectation (Figures 4B, S17 and S18).

Finally, we set out to look at the product specificity of MysDs with other patterns at the two residues. Given that MysD with an L–A pattern and previously characterized *Microcystis aeruginosa* PCC 7806 MysD with a C–A pattern preferentially producing shinorine,^17^ we hypothesized that MysDs with an X–A pattern (except for F–A) preferentially produce shinorine. We expressed MysDs from *M. aeruginosa* PCC 7806 (C–A) and *Coleofasciculus chthonoplastes* PCC 7420 (C–A) and *Cyanothece* sp. BG0011 (Y–A) and *Crocosphaera chwakensis* CCY0110 (Y–A) in SC.L26 and all of them produce predominantly shinorine, suggesting a relationship between A in the second residue and shinorine product specificity with the exception of F–A (Figures 4B, S19–S22). Similarly, we hypothesized that MysDs with an X–S pattern (except for L–S) preferentially produce porphyra-334. We expressed MysDs from *Nostoc* sp. PA-18-2419 (Y–S) and *Nostoc* sp. NIES-4103 (Y–S), *Calothrix elsteri* CCALA 953 (A–S) and *Calothrix* sp. PCC 6303 (A–S), *Chamaesiphon polymorphus* CCALA 037 (M–S) and *Chamaesiphon minutus* PCC 6605 (M–S) in SC.L26 and all of them produce predominantly porphyra-334, suggesting a relationship between S in the second residue and porphyra-334 product specificity with the exception of L–S (Figures 4B, S23–S28).

To validate that product specificity arises from key residue pattern rather than sequence similarity of whole enzymes, we constructed the phylogenetic tree of our characterized MysDs (Figure S6). Sporadic distribution of MysDs with different product specificities throughout the tree suggests independence of product specificity from sequence similarity.

Discovery of the relationship between the pattern of key residues and product specificity enables us to screen for more specific shinorine and porphyra-334 producers with minimal side-product produced. Among all MysDs we characterized, MysD from *M. aeruginosa* PCC 7806 (C–A) shows the highest shinorine-to-porphyra-334 ratio, while MysD from *Nostoc* sp. PA-18-2419 (Y–S) shows the highest porphyra-334-to-shinorine ratio (Figure S4). These novel MysD orthologues hold significant potential in the large-scale industrial synthesis of MAAs.

## Conclusions

In this study, we explored strategies to enable the efficient and specific production of MAAs through pathway engineering in *S. cerevisiae*. Through introduction of the xylose utilization pathway and multiple copies of MAA biosynthesis pathway genes and knock-down of endogenous competing pathways, we developed a yeast strain capable of producing MG, the precursor of all disubstituted MAAs. This yeast strain allows us to engineer shinorine and porphyra-334 production through expressing wild-type and mutant MysDs and explore MysD’s residue pattern-product specificity relationship. Using a structure-guided mutagenesis approach, we corroborated on Kim et al., 2023’s result on the role of omega-loop in product specificity determination and took a step further, narrowing it down to the identity of only two residues. The residue pattern-product specificity correlation was supported by pattern-guided selection and characterization of MysD homologues, suggesting that direct interactions of residues with the seryl or threonyl moieties of the intermediate is a potential mechanism of product specificity. Hence, we can exploit the pattern of these two residues to predict, fine-tune, and alter MysD product specificity. Our finding provides guidance for selecting uncharacterized MysD candidates for further screening and sheds light on the rational modifications on MysD that enables it to conjugate novel amino acid substrates.

## Materials and Methods

### Protein Structure Prediction and Molecular Docking

3D structures of *Np.*MysD and *Nl.*MysD were predicted using ColabFold online server.^19^ Ligand model was constructed and visualized using ChemOffice Professional, Version 20.0. Structural alignment and visualization of proteins were performed using PyMOL v2.0. AutoDockTools 1.5.7 was used to preprocess enzyme and ligand files, and Autodock Vina 1.2.0 was used to dock ligand into enzyme active sites.^20−22^ The molecular conformations with the lowest kilocalories per mole were used for the subsequent analysis.

### Multi-Sequence Alignment and the Construction of Phylogenetic Tree

Amino acid sequences of *Np.*MysD and *Nl.*MysD were acquired from the NCBI Nucleotide Database (Accession codes: ACC83902.1, BAY79928.1).^23^ The sequences were imported into MEGA v11.0, and a two-sequence alignment was performed using the MUSCLE algorithm with default parameters.^24^

The amino acid sequences of the 20 cyanobacterial MysD orthologs were either acquired from the OrthoDB v11 Database or the NCBI Protein Database.^23,25^ The sequences were imported into MEGA v11.0 together with the sequence of *Np.*MysD and *Nl.*MysD and aligned using the MUSCLE algorithm with default parameters.^24^ Maximum-Likelihood tree was constructed based on this 22-sequence alignment by IQ-TREE2, using model JTTDC-Mut + I + G with 1000 rounds of UFBoot.^26,27^ Phylogenetic tree was visualized and annotated using iTOL v6.35.^28^

### Engineering MAA Production Yeast Strains

Coding sequences of all genes used in this study (*XYL1–3, Np.mysA-D, Nl.mysD*, 20 newly characterized *MysDs*) with BsaI overhangs were synthesized from BGI Genomics (Shenzhen, China) and assembled onto an intermediate cloning vector that contains a pair of Golden Gate-compatible BsaI sites flanked with M13 sequencing primer binding sites (Table 1, row 5–12). They were assembled onto link-021, link-022, link-024, and link-027, which contain promoter and terminator sequences upstream and downstream of the insertion site, using the NEBridge Golden Gate Assembly Kit (BsaI-HF v2) (Table 1). The 20 μL system containing 1 μL of BsaI-HF v2, 1 μL of T4 DNA ligase, 2 μL of T4 Ligase buffer, and the DNA mixture (target vector: insert molar ratio = 1:2) was incubated for 30 cycles of alternating 5 min 37 °C–5 min 16 °C reaction cycle. Assembly reactions were transformed into *E. coli* (DH5-Alpha), and assembled plasmids were extracted and verified by Sanger sequencing (BGI Genomics, Shenzhen, China).

**Table 1:** Plasmids Used in This Study

plasmid	description	reference
links021	2U-URA3-LS-P_*TDH3*_-BsaI-T_*TDH1*_-R1	this study
links024	2U-URA3-L1-P_*PGK1*_-BsaI-T_*PGK1*_-R2	this study
links027	2U-URA3-L3-P_*TEF2*_-BsaI-T_*SSA1*_-RE	this study
links022	2U-URA3-P_*PGK1*_-BsaI-T_*CYC1*_-R2	this study
type3-*XYL1*	M13F-BsaI-*XYL1*-BsaI-M13R	this study
type3-*XYL2*	M13F-BsaI-*XYL2*-BsaI-M13R	this study
type3-*XYL3*	M13F-BsaI-*XYL3*-BsaI-M13R	this study
type3-*Np.mysA*	M13F-BsaI-*Np.mysA*-BsaI-M13R	this study
type3-*Np.mysB*	M13F-BsaI-*Np.mysB*-BsaI-M13R	this study
type3-*Np.mysC*	M13F-BsaI-*Np.mysC*-BsaI-M13R	this study
type3-*Np.mysD*	M13F-BsaI-*Np.mysD*-BsaI-M13R	this study
type3-*Nl.mysD*	M13F-BsaI-*Nl.mysD*-BsaI-M13R	this study
type9K-*XYL1*-*XYL2*-*XYL3*	P_*TDH3*_-*XYL1*-T_*TDH1*_-P_*PGK1*_-*XYL2*-T_*CYC1*_-P_*TEF2*_-*XYL3*-T_*SSA1*_	this study
pCRCT-*TAL1*	ura3, P_*SNR52*_-*TAL1*-T_*sup4*_, P_*TEF1*_-*CAS9*-T_*RPR1*_ flanked by *TAL1* upstream and downstream	this study
pCRCT-*HIS3*	ura3, P_*SNR52*_-*HIS3*-T_*SUP4*_, P_*TEF1*_-*CAS9*-T_*RPR1*_	this study
pCRCT-*308a*	ura3, P_*SNR52*_-*308a*-T_*SUP4*_, P_*TEF1*_-*CAS9*-T_*RPR1*_	this study
pCRCT-*YP*	ura3, P_*SNR52*_-*YPRCd15c*-T_*SUP4*_, P_*TEF1*_-*CAS9*-T_*RPR1*_	this study
pCRCT-*NQM1*	ura3, P_*SNR52*_-*NQM1*-T_*SUP4*_, P_*TEF1*_-*CAS9*-T_*RPR1*_	this study

*TAL1* and *NQM1* knockout and *XYL1–3* and *mysA-C* genomic insertion were implemented using CRISPR-Cas9.^29^ For genomic insertion, inserts flanked with homology arms were linearized from the assembled plasmids by using PCR. *S.cerevisiae* strains were then chemically transformed with pCRCTs along with linear fragments containing insertion sequences^30^ (Table 1). For chemical transformation, competent *S.cerevisiae* cells pelleted from 5 to 10 mL expansion culture (end OD600 = 0.5–0.8) were mixed with a 360 μL lithium acetate transformation mixture (250 μL of 50% PEG3350, 36 μL of 1 M LiAc, 10 μL of 2 mg/mL salmon sperm ssDNA, 250 ng of pCRCT and 2000 ng of insertion fragments) under 42 °C for 40 min. Following uracil auxotrophy selection, colony PCR and Sanger sequencing were applied for verification (BGI Genomics, Shenzhen, China).

### Point-Mutations and Omega-Loop Switching

Primers were designed for site-directed mutagenesis (Table S4). Mutagenesis PCR using primers with mutation sites at overlapping 5′ overhangs and NEB Q5 High Fidelity DNA polymerase followed by Gibson Assembly was performed to introduce mutations on plasmids. Mutations were verified by Sanger sequencing (BGI Genomics, Shenzhen, China).

### *S. cerevisiae* Culture and Metabolite Extraction

Strains used in this study are given in Table 2. Liquid SC-Ura medium (YNB 1.7 g/L, Amino Acid Supplement Mixture (-His, -Trp, -Leu, -Ura) 1.4 g/L, Ammonium sulfate 5g/L, Leu 380 mg/L, Trp 76 mg/L, His 76 mg/L, and 2% glucose) was used for culturing *S. cerevisiae*. Cultures were grown in 100 mL of SC-Ura medium in 250 mL Erlenmeyer flasks at 30 °C with 200 rpm shaking for 48 h. Suspension of culture was harvested and subjected to two freeze–thaw cycles (frozen at −80 °C and thawed at 25 °C) before being centrifuged at 10000 rpm for 10 min. Supernatant was then harvested and filtered with a 0.22 μm filtration membrane and stored at −20 °C before LC-HRMS analysis.

**Table 2:** Strains Used in This Study

strain	genotype	reference
CEN.PK2	MATa *ura*3-52 *trp*1-289 *leu*2-3112 *his3*Δ1 *MAL*2-8^c^*SUC2*	EUROSCARF
SC.L1	CEN.PK2 *tal1*Δ	this study
SC.L2	SC.L1 *HIS3*::P_*TDH3*_-XYL1-T_*TDH1*_-P_*PGK1*_-XYL2-T_*CYC1*_-P_*TEF2*_-XYL3-T_*SSA1*_	this study
SC.L3	SC.L2 308::P_*TDH3*_-np.mysA-T_*TDH1*_-P_*PGK1*_-np.mysB-T_*PGK1*_	this study
SC.L6	SC.L3 *NQM1*::P_*TDH3*_-np.mysB-T_*TDH1*_-P_*PGK1*_-np.mysA-T_*PGK1*_	this study
SC.L26	SC.L6 YP::P_*TDH3*_-np.mysC-T_*TDH1*_	this study

### LC-HRMS Analytical Methods

LC-HRMS analysis was performed on a UHPLC Vanquish flex system, equipped with a variable wavelength detector (Thermo Fisher Scientific, Bremen, Germany), coupled to a Q-Exactive Plus mass spectrometer (Thermo Fisher Scientific, Bremen, Germany) and operated in the positive (ESI^+^) and negative (ESI^–^) electrospray ionization modes (one run for both modes). The system was controlled by an Xcalibur 4.2 (Thermo Fisher Scientific). The HESI (heated electrospray ionization) source used was a spray voltage of 3.0 kV for ESI^+^ and −2.8 kV for ESI^–^, capillary temperature of 350 °C, heater temperature of 350 °C, sheath gas flow of 50 arbitrary units (AU), auxiliary gas flow of 15 AU, sweep gas flow of 2 AU, and S-Lens RF level of 50%. During the full-scan acquisition, which ranged from *m*/*z* 100 to 800, the instrument operated at 70,000 resolution at *m*/*z* = 200, with an automatic gain control (AGC) target of 3 × 10^6^ and a maximum injection time (MIT) of 100 ms. For MS2 analysis, the isolation window was set at 4.0 *m*/*z*, and the instrument was operated at 17,500 resolution at *m*/*z* = 200, with an AGC target of 1 × 10^5^, MIT of 50 ms, and stepped NCE of 30, 40, and 50 eV with N2 as collision gas.

Chromatographic separation was carried out with a UPLC HSS T3 column (2.5 μm, 100 × 2.1 mm) at a temperature of 25 °C. The mobile phases consisted of 0.1% formic acid in water (A) and acetonitrile (B). The elution gradient was as follows: 0–5 min, 1% B; 5–7 min, 1–30% B; 7–9 min, 30–99% B; 9–10 min, 99% B; 10–10.1 min, 99–1% B; and 10.1–15 min, 1% B, at a flow rate of 0.2 mL/min. The injection volume was 1 μL. UV absorption was set at a fixed wavelength of 334 nm.

### Metabolite Content Quantification

Shinorine and porphyra-334 standard samples were acquired from Beijing Yiming Fuxing Biological Technology Co., Ltd. Concentrations of shinorine and porphyra-334 were calculated by comparing peak areas produced by each sample with the peak areas of 0.1 mg/mL external standards according to the following equation:
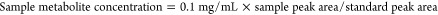


## Supporting Information Available

Full names of gene and protein abbreviations, NCBI accession codes of genes and proteins used in this study, sgRNA and primer sequences used in this study, and raw LC-HRMS graphs (PDF)
