# Supplementary material for: Decoding Specificity of Cyanobacterial MysDs in Mycosporine-like Amino Acid Biosynthesis through Heterologous Expression in Saccharomyces cerevisiae
Source: ACS Omega. 2025 Mar 28;10(13):13664–73. doi: 10.1021/acsomega.5c01035 (PMC11983340; doi:10.1021/acsomega.5c01035)

Decoding Specificity of Cyanobacterial MysDs in Mycosporine-Like Amino Acid Biosynthesis through Heterologous Expression in *Saccharomyces cerevisiae*

Xiaoyou Zheng[1], Peifeng Xie[2†], Andrew Chen Cai[2†], Yuze Jiang[2], Sirui Huang[3], Xiaochong Ma[2], Honghao Su[4*], Boxiang Wang[2*]

†: Authors contributed equally

*: Corresponding authors

Corresponding emails: Honghao.Su@earlham.ac.uk, wangboxiang@link-spider.com

1. Churchill College, University of Cambridge, Storey's Way, Cambridge, United Kingdom, CB3 0DS
2. LINK SPIDER Co., Ltd., 11 Langshan Rd, Nanshan District, Shenzhen, China, 518000
3. Thurgood Marshall College, University of California, San Diego, 9500 Gilman Dr., La Jolla, California, United States
4. Earlham Institute, Norwich Research Park, Norwich, United Kingdom, NR4 7UZ

Supplementary Materials

Glossary. Abbreviations and full names of the metabolites involved in this study.

Abbreviation	Full Name
G6P	Glucose 6-phosphate
F6P	Fructose 6-phosphate
F1,6P	Fructose 1,6-bisphosphate
G3P	Glyceraldehyde 3-phosphate
DHAP	Dihydroxyacetone phosphate
1,3PG	1,3-Bisphosphoglycerate
6PGL	6-Phosphogluconolactone
6PG	6-Phosphogluconate
Ru5P	Ribulose 5-phosphate
R5P	Ribose 5-phosphate
X5P	Xylulose 5-phosphate
S7P	Sedoheptulose 7-phosphate
E4P	Erythrose 4-phosphate
DDG	Desmethyl 4-deoxygadusol
4-DG	4-Deoxygadusol
MG	Mycosporine-glycine

Table S1. Genes used in metabolic engineering.

gene name	ACCESSION	organism name
XYL1	XP_001385181	*Scheffersomyces stipitis* CBS 6054
XYL2	XP_001386982	*Scheffersomyces stipitis* CBS 6055
XYL3	XP_001387325	*Scheffersomyces stipitis* CBS 6056
mysA(DDG)	WP_012411849	*Nostoc punctiforme*
mysB(O-MT)	WP_012411848	*Nostoc punctiforme*
mysC(AGL)	WP_012411847	*Nostoc punctiforme*
Np.mysD	WP_012411846	*Nostoc punctiforme*
Nl.mysD	BAY79928	*Nostoc linckia* NIES-25

Table S2. sgRNA Sequences.

Target site	Guide Sequence
TAL1	ACTGTCGTTGTTGCCGACAC
HIS3	TGCCTCGCAGACAATCAACG
308a	CACTTGTCAAACAGAATATA
Nqm1-1	GATCAAGATAGCTTCTACGT
YPRCd15c	AATCCGAACAACAGAGCATA

Table S3. Novel *mysDs* from 20 cyanobacteria strains.

gene name	ACCESSION	organism name	Source
CA-267872	WKX64033.1	*Microcystis aeruginosa PCC 7806*	NCBI Proteins
FS-450202	BBB38339.1	*Nostoc commune KU002*	NCBI Proteins
LA-76335	WP_114083456	*Nostoc sp.* ATCC 53789	OrthoDB v11
LA-46234	WP_015081019	*Anabaena sp.* 90	OrthoDB v11
FS-1932621	WP_086687004	*Nostoc sp.* T09	OrthoDB v11
FS-1628751	PHK43867	*Nostoc linckia* z16	OrthoDB v11
FS-446679	WP_118166108	*Nostoc sphaeroides*	OrthoDB v11
FA-102232	WP_006527735	*Gloeocapsa sp.* PCC 73106	OrthoDB v11
CA-118168	WP_006100251	*Coleofasciculus chthonoplastes* PCC7420	OrthoDB v11
AS-1170562	WP_015199129	*Calothrix sp.* PCC 6303	OrthoDB v11
AS-987040	WP_095720873	*Calothrix elsteri* CCALA 953	OrthoDB v11
YS-2575443	WP_138500196	*Nostoc sp.* PA-18-2419	OrthoDB v11
YS-2005458	BAZ51206	*Nostoc sp.* NIES-4103	OrthoDB v11
YA-2082950	WP_107668801	*Cyanothece sp.* BG0011	OrthoDB v11
YA-391612	WP_008274182	*Crocosphaera chwakensis* CCY0110	OrthoDB v11
MS-2107692	PSB40329	*Chamaesiphon polymorphus* CCALA 037	OrthoDB v11
MS-1173020	WP_015160004	*Chamaesiphon minutus* PCC 6605	OrthoDB v11
LS-179408	WP_015177382	*Oscillatoria nigro-viridis* PCC 7112	OrthoDB v11
LA-457944	WP_069074327	*Nostoc sp.* KVJ20	OrthoDB v11
LA-163908	WP_016949469	*Anabaena sp.* PCC 7108	OrthoDB v11

Table S4. Primers for omega-loop exchanging and point-mutagenesis.

Template	Primer Pair	Sequence (5'-3')	Amplicon
links024-*Np.mysD*	*Np.*Loop-A-F	GTTTCGCTGCCAAGGGTAACAACAAATC TTGGATTTTAGACCCTAACGACC	Fragment-1.
	*Np.*Loop-A-R	CATCAGTTTTCTTCAACTTGTCTGCGTAG GTACGGATAGGTTTATCGTGTGG	
Fragment-1	*Np.*Loop-B-F	AAAACTGATGACGGTTCTTTAGGTTTCG CTGCCAAGGGTA	Fragment-1 with two homology arms. Became links024-*Np.mysD-Nl.*Loop after Gibson assembly.
	*Np.*Loop-B-R	AGCGAAACCTAAAGAACCGTCATCAGTT TTCTTCAACTTGTCTG	
links024-*Np.mysD*	*Np.*LmutF-F	GCGACTTGCATTTCACTGCTAAAGATAA TATCAAGGCTTGG	Fragment-2. Became links024-*Np.mysD*-L249F after Gibson assembly.
	*Np.*LmutF-R	TATTATCTTTAGCAGTGAAATGCAAGTC GCCATCGTCA	
links024-*Np.mysD*	*Np.*AmutS-F	AGATAATATCAAGTCTTGGATTTTAGAC CCTAACGACC	Fragment-3. Became links024-*Np.mysD*-A257S after Gibson assembly.
	*Np.*AmutS-A-R	GGGTCTAAAATCCAAGACTTGATATTAT CTTTAGCAGTCAAATGCAAG	
links024-*Np.mysD*-L249F	*Np.*AmutS-F	AGATAATATCAAGTCTTGGATTTTAGAC CCTAACGACC	Fragment-4. Became links024-*Np.mysD*-L249F-A257S after Gibson assembly.
	*Np.*AmutS-B-R	GGGTCTAAAATCCAAGACTTGATATTAT CTTTAGCAGTGAAATGCAAG	
links024-*Nl.mysD*	*Nl.*Loop-A-F	GCATTTGACTGCTAAAGATAATATCAAG GCTTGGATTTTGGACCCAAACGA	Fragment-5.
	*Nl.*Loop-A-R	CGTCAGTTTGTTGGAGTTTATCAGCATA GTTTCTGATTGGTTTTTCTTGAGAGTCG	
Fragment-5	*Nl.*Loop-B-F	CAAACTGACGATGGCGACTTGCATTTGA CTGCTAAAGATAATATCAAGG	Fragment-5 with two homology arms. Became links024-*Nl.mysD-Np.*Loop after Gibson assembly.
	*Nl.*Loop-B-R	AGTCAAATGCAAGTCGCCATCGTCAGTT TGTTGGAGTTTATCAG	
links024-*Nl.mysD*	*Nl.*FmutL-F	GTTCTTTAGGTTTAGCTGCCAAGGGTAA CAACAA	Fragment-6. Became links024-*Nl.mysD*-F247L after Gibson assembly.
	*Nl.*FmutL-R	CCCTTGGCAGCTAAACCTAAAGAACCGT CATCAGTT	
links024-*Nl.mysD*	*Nl.*SmutA-F	AAGGGTAACAACAAAGCTTGGATTTTGG ACCCAAACGA	Fragment-7. Became links024- *Nl.mysD*-S255A after Gibson assembly.
	*Nl.*SmutA-R	GTCCAAAATCCAAGCTTTGTTGTTACCC TTGGCAGC	
links024-*Nl.mysD*-F247L	*Nl.*SmutA-F	AAGGGTAACAACAAAGCTTGGATTTTGG ACCCAAACGA	Fragment 8. Became links024-*Nl.mysD*-F247L-S255A after Gibson assembly.
	*Nl.*SmutA-R	GTCCAAAATCCAAGCTTTGTTGTTACCC TTGGCAGC	

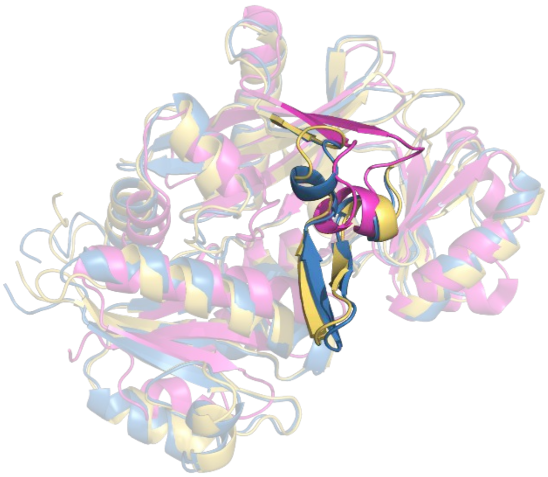

Figure S1. Structural alignment of the predicted structural model of *Nl.*MysD (blue) and the predicted structural model of *Np.*MysD (yellow) with the crystal structure (magenta) of Escherichia coli d-alanine-d-alanine ligase (2DLN). The omega-loops are highlighted with opacity while the rest of the structures are transparent. Water and ligand molecules from the original PDB file of 2DLN are removed.

```
NpMysD/1-348   15 DKFYCDLSRLYAQMFCAHKARAKAIVEAAGVKVPRGELLRQGDIPTITPPAVVKPVSSDNSLGVVLVKDVTEYDAALKKAFEYASEVIVEAFIELGR 202
NlmysD/1-368   15 DDFYCDLSRLYAQMFCAHKARTKAIVEAAGVKVPRGEVLRRGDVPTITPPVVIKPVSSDNSLGVTLVKDAAEYEAALEKAFEHGDEAIVETFIE GR 200

NpMysD/1-348  203 EVRCGIIVKDGELIGLPLEEYLVDPHDKPIRNYADKLQQTDDGDLHLTAKDNIKAWILDPNDPITQKVQQVAKRCHQALGCRHYSLFDFRIDPKGQP 299
NlmysD/1-368  201 EVRCGIIVKDGELIGLPLEEYLIDSQEKPIRTYADKLKKTDDGSLGFAAKGNNKSWILDPNDPITQKVQEVAKKCHQALGCRHYSLFDFRIDSQGQP 297

NpMysD/1-348  300 WFLEAGLYCSFAPKSVISSMAKAAGIPLNDLLITAINETLGSNKKVLQN------------------------                          348
NlmysD/1-368  298 WFLEAGLYCSFAPKSVISSMAKAVGIPLNELLTIAIAETLGSNK--YSDRISVVEINEPSKTPRKERELSQMI                          368
```

Figure S2. The complete version of the aa sequence alignment between *Np.*MysD and *Nl.*MysD. The identical residues within 5 angstroms from the docked reaction intermediate (P-MG-Ser) are colored orange. The two critical aa residues are highlighted with yellow (*Np.*MysD) or cyan (*Nl.*MysD). The 26-aa long fragment of the omega-loop (pink) contains all the variable aa residues within 8 angstroms.

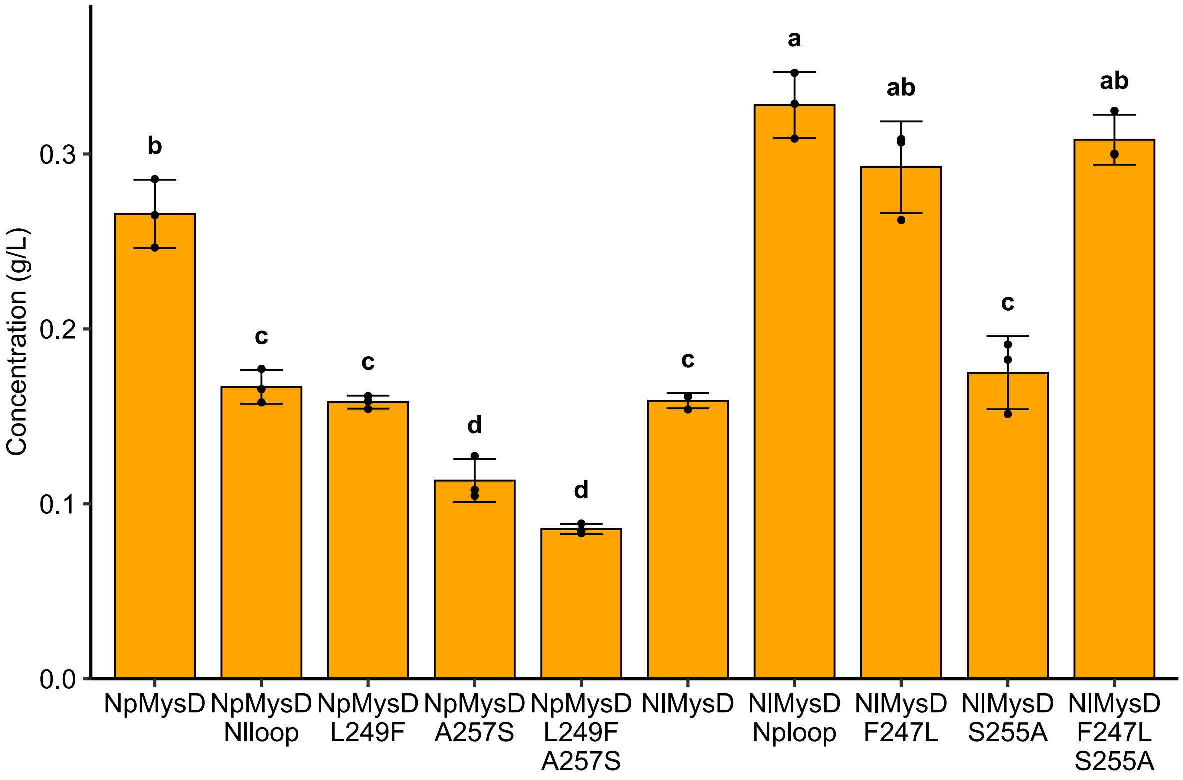

Figure S3. Quantification of total enzyme activity by comparing the sum of shinorine and porphyra-334 peak area amongst these yeast strains. Heights of the bars represent their means and the error bars represent their standard deviations (n=3 biological replicates). One-way ANOVA was performed to compare the total amount of shinorine and porphyra-334 amongst different samples and two samples that do not share the same letter are significantly different from each other ($p<0.05$).

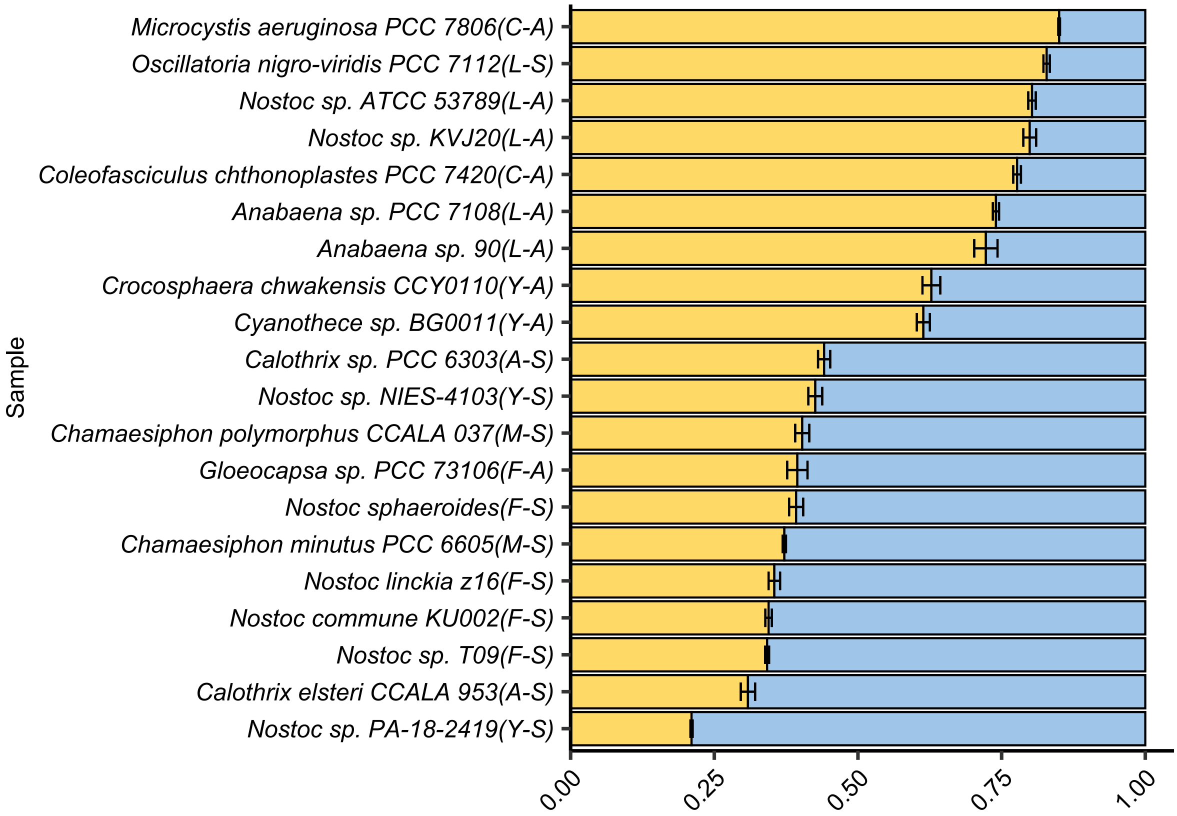

Figure S4. Shinorine (yellow) : porphyra-334 (blue) ratio from LC-HRMS peak area analysis by yeast strains expressing selected MysDs. Heights of the bars represent their means and the error bars represent their standard deviations (n=3 biological replicates).

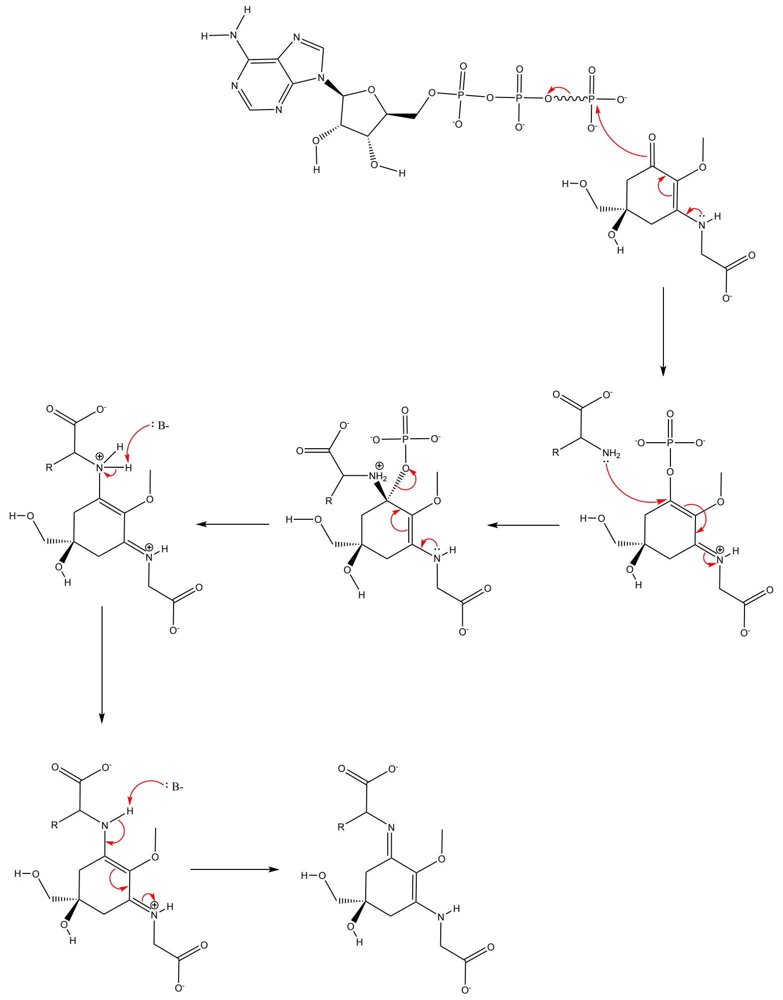

Figure S5. Proposed step-wise mechanism of the reaction catalyzed by MysDs. The process is initiated by the transfer of the terminal phosphate group from ATP to the C1 position of MG. The amino acid substrate is then conjugated with MG via a nucleophilic attack on C1. Through series of molecular rearrangements and deprotonations, the reaction is completed with the removal of the phosphate group.

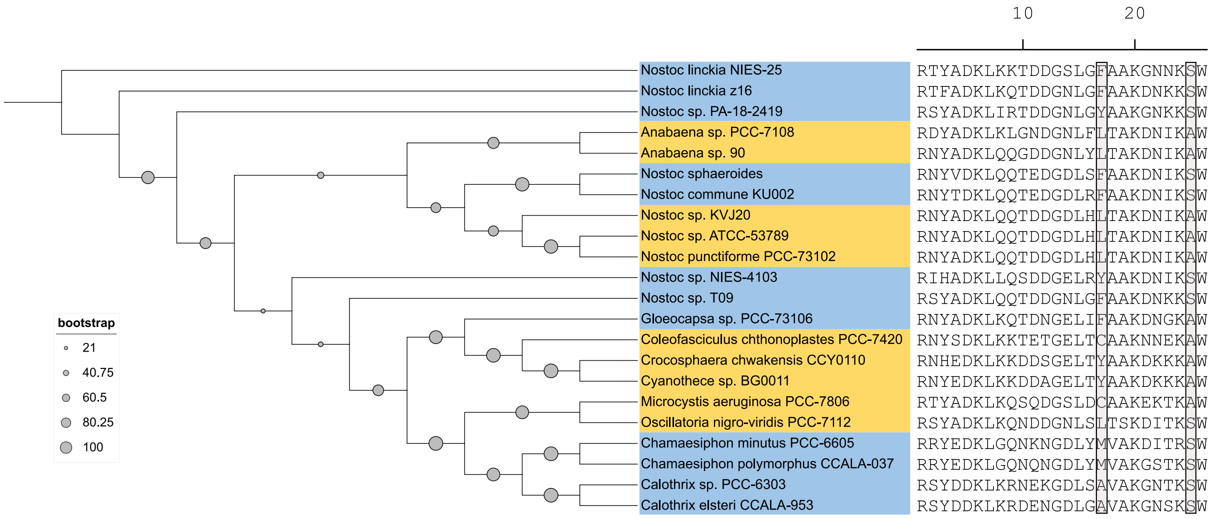

Figure S6. The Maximum-Likelihood (ML) phylogenetic tree of the 20 MysD orthologs. The tree was constructed using whole-sequence alignment and the 26-aa omega-loop fragments are shown. The bootstrap value of each node in the tree is demonstrated. The colors superimposed on the name of the strains indicate their product-specificity analyzed with LC-HRMS peak area. Blue indicates porphyra-334 preference, while yellow indicates shinorine preference. The two critical residues are labeled with grey boxes.

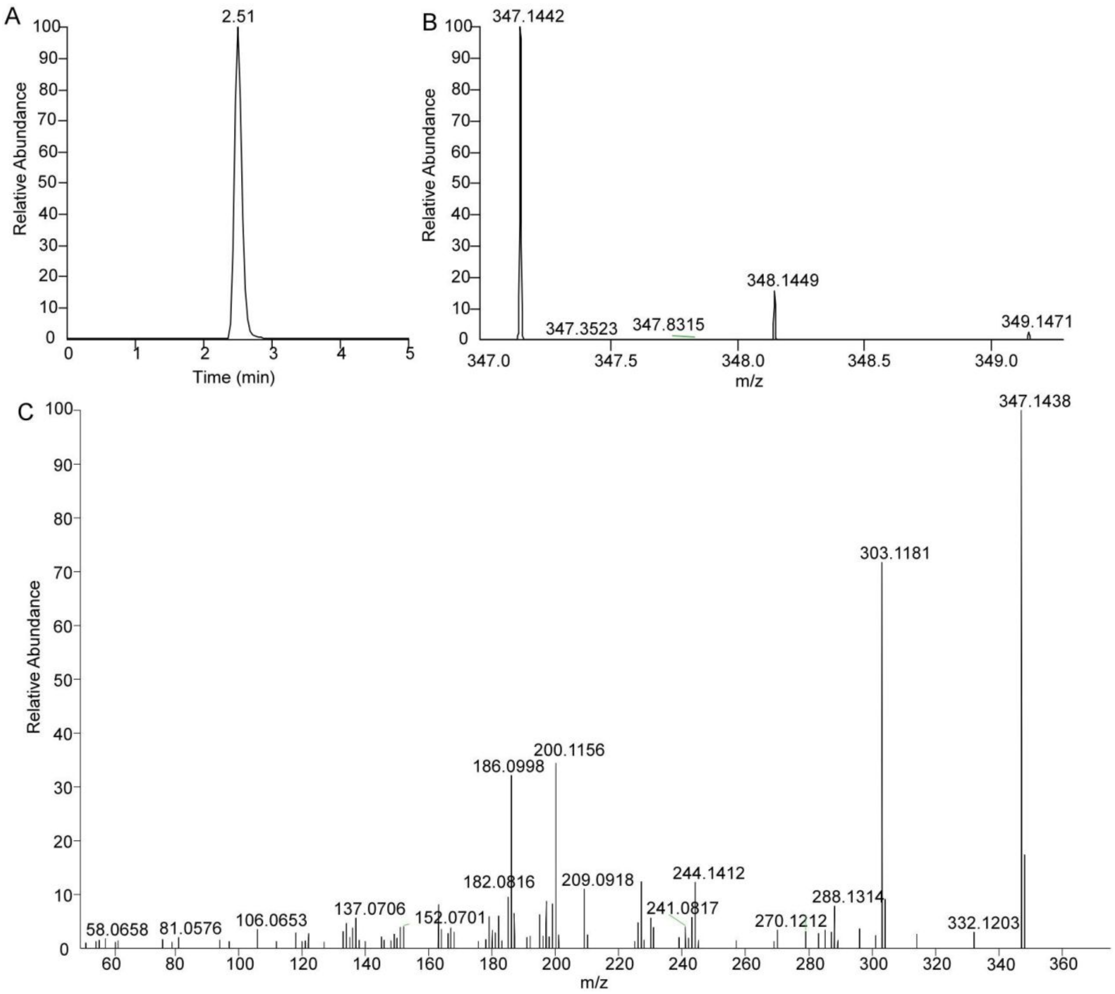

Figure S7. The extracted chromatogram peak, MS[1] and MS/MS spectra of porphyra-334 for the characterization of the 20 MysD orthologs (A-C).

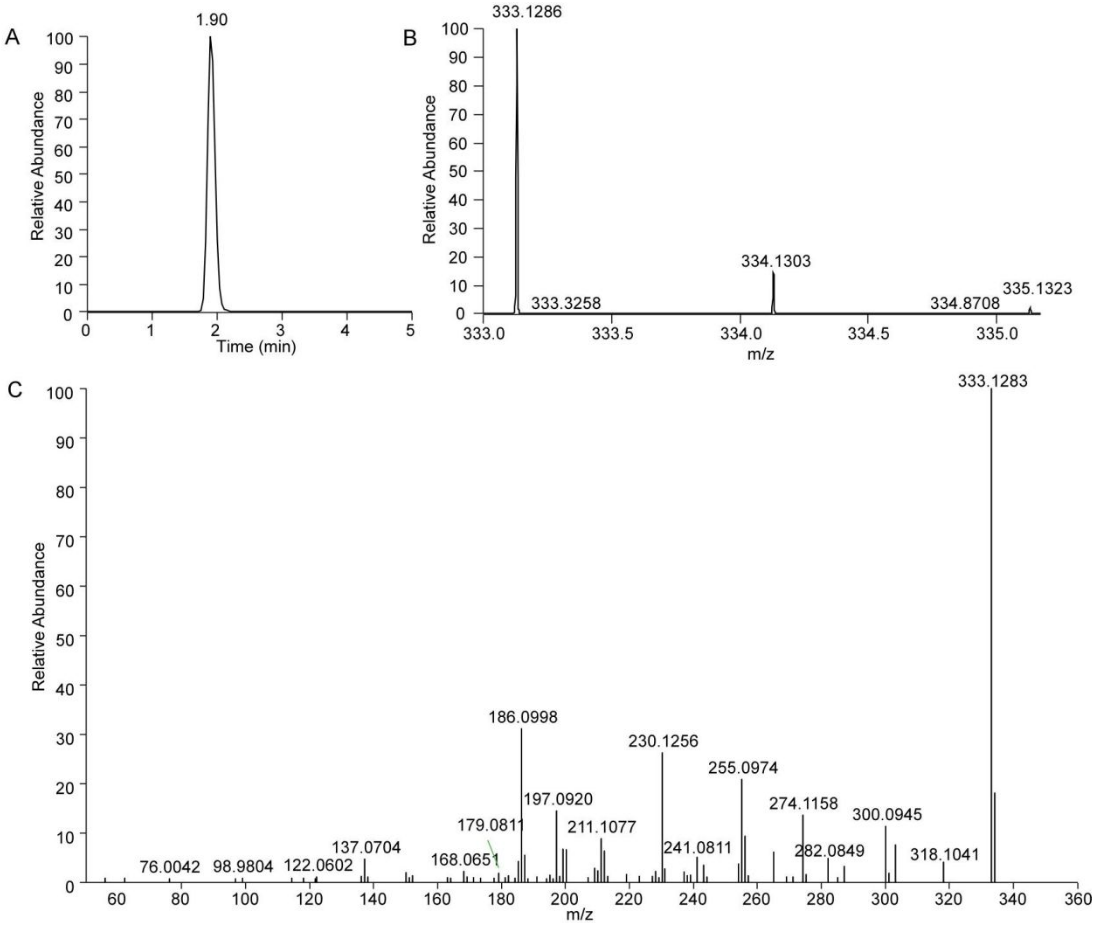

Figure S8. The extracted chromatogram peak, MS^1 and MS/MS spectra of shinorine for the characterization of the 20 MysD orthologs (A-C).

Figure S9-S28. Chromatogram peaks of shinorine and porphyra-334 in the 20 MysD orthologs.

S9. Anabaena sp. 90(L-A)

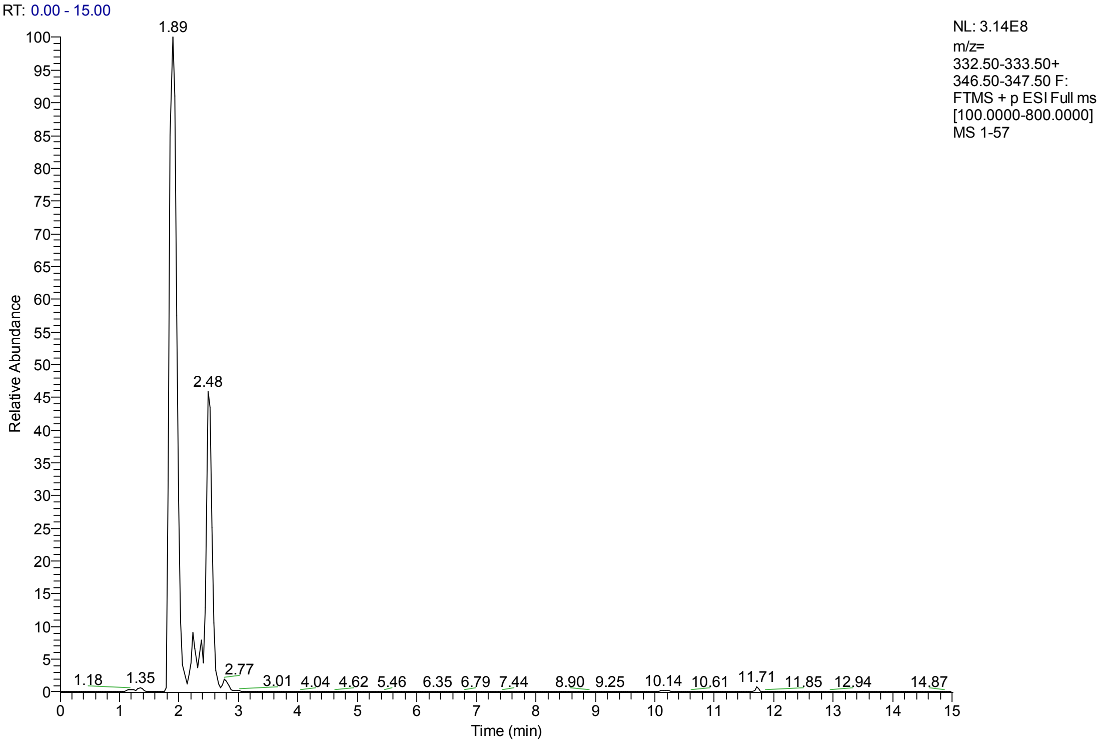

S10. Anabaena sp. PCC 7108(L-A)

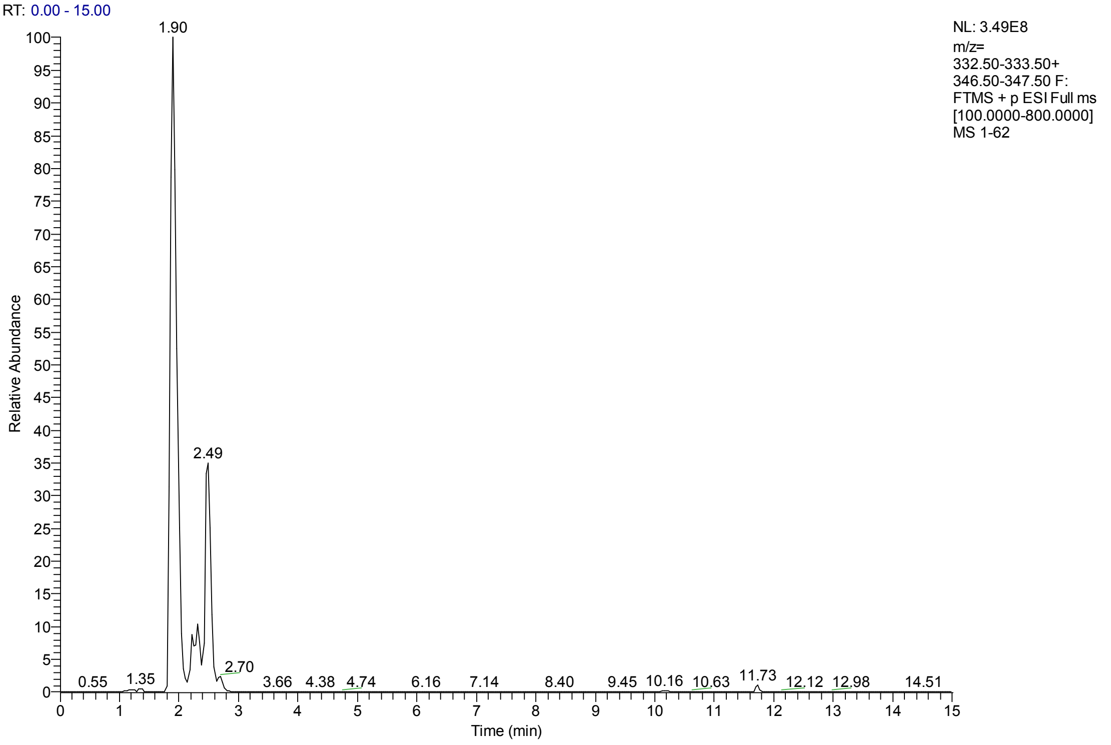

S11. Nostoc sp. KVJ20(L-A)

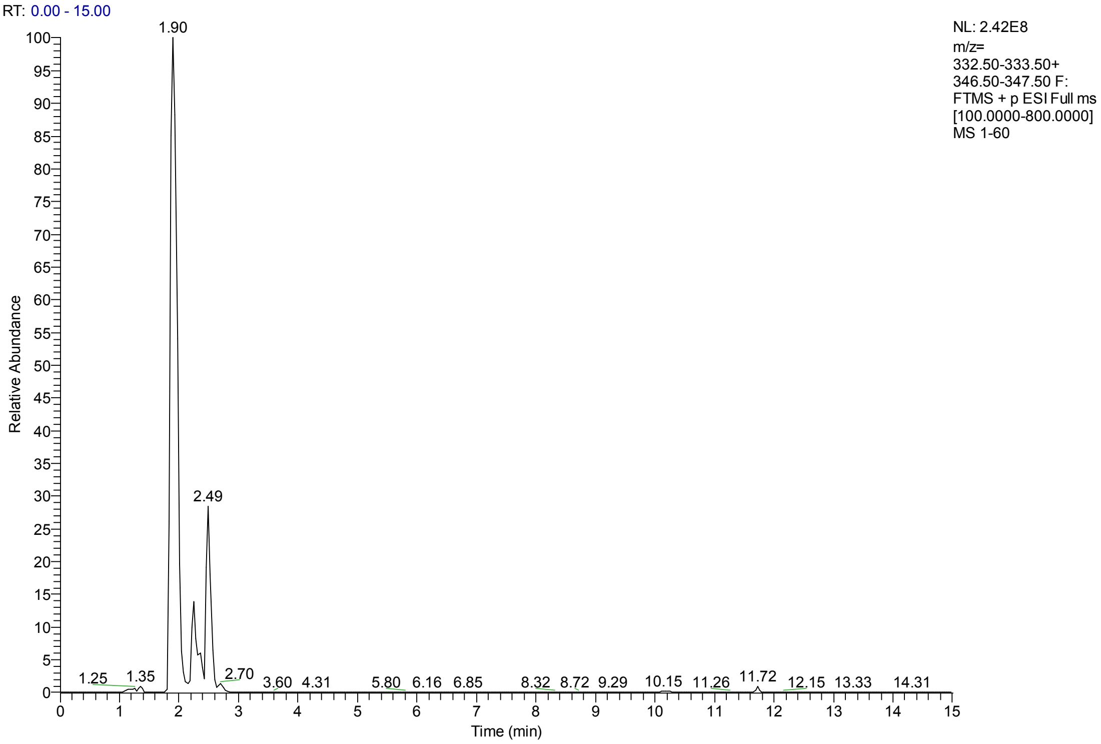

S12. Nostoc sp. ATCC 53789(L-A)

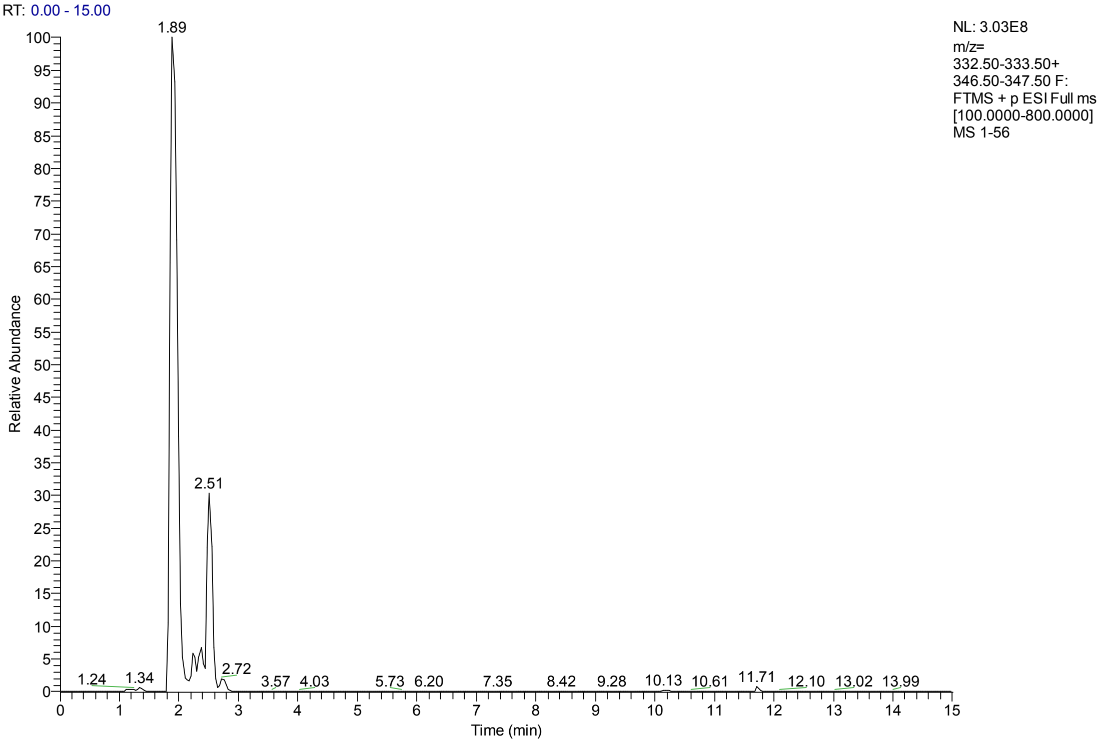

S13. Nostoc linckia z16(F-S)

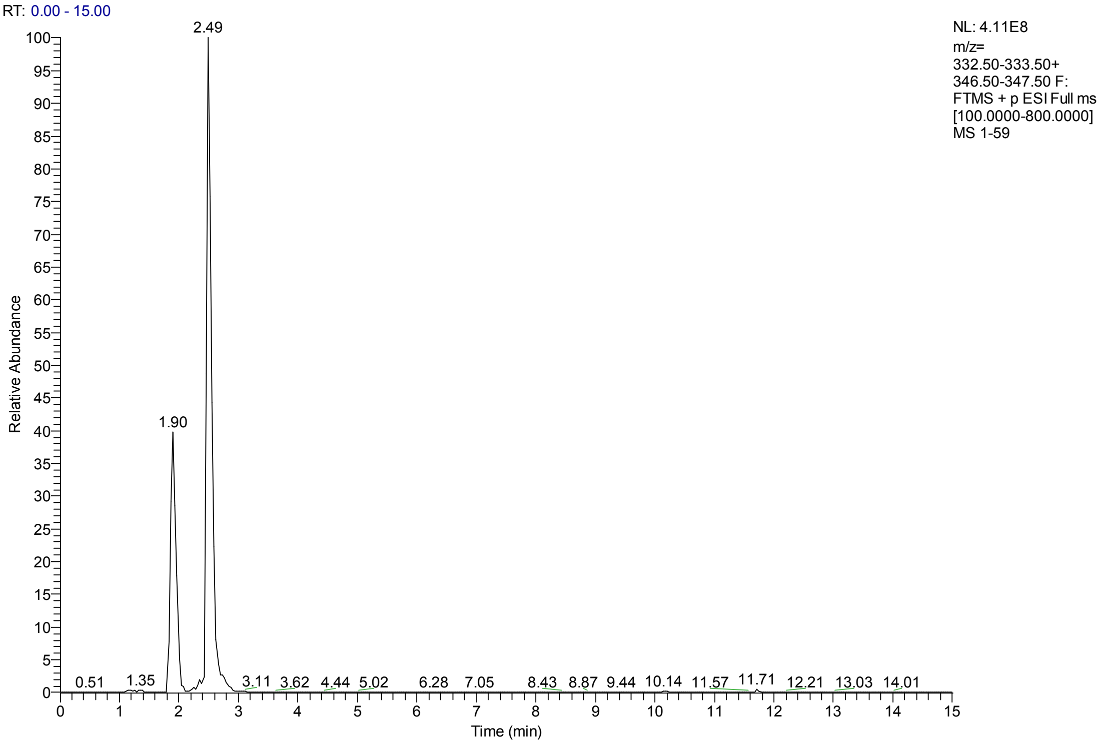

S14. Nostoc sp. T09(F-S)

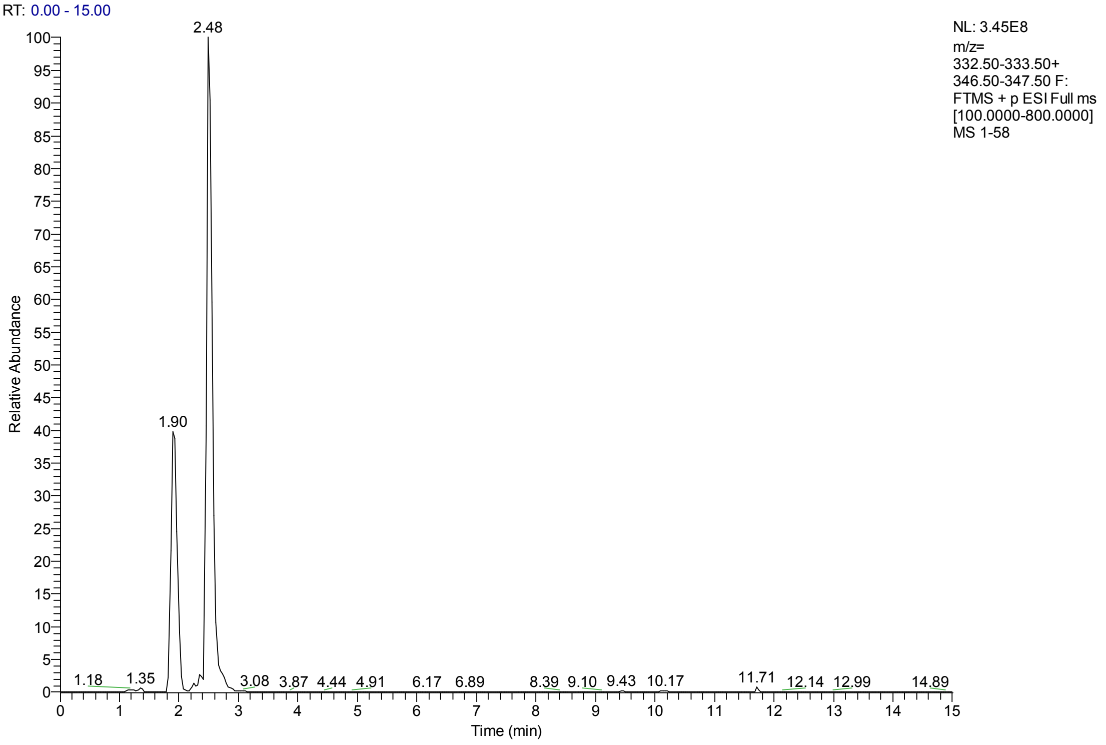

S15. Nostoc commune KU002(F-S)

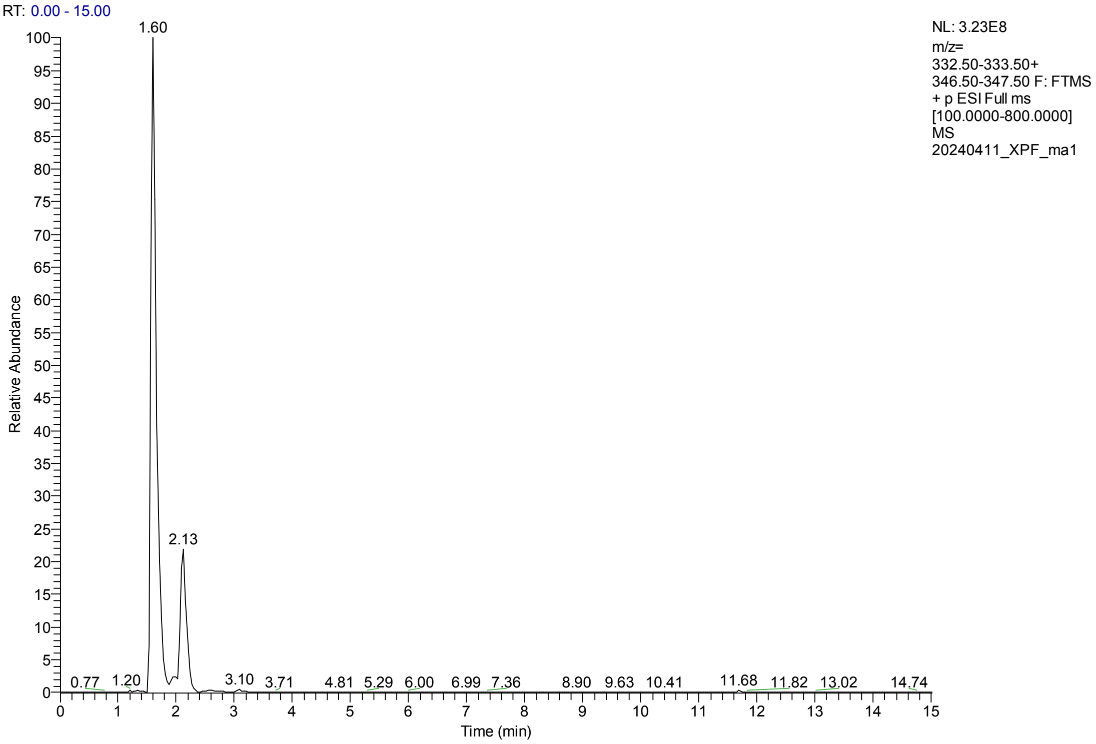

S16. Nostoc sphaeroides(F-S)

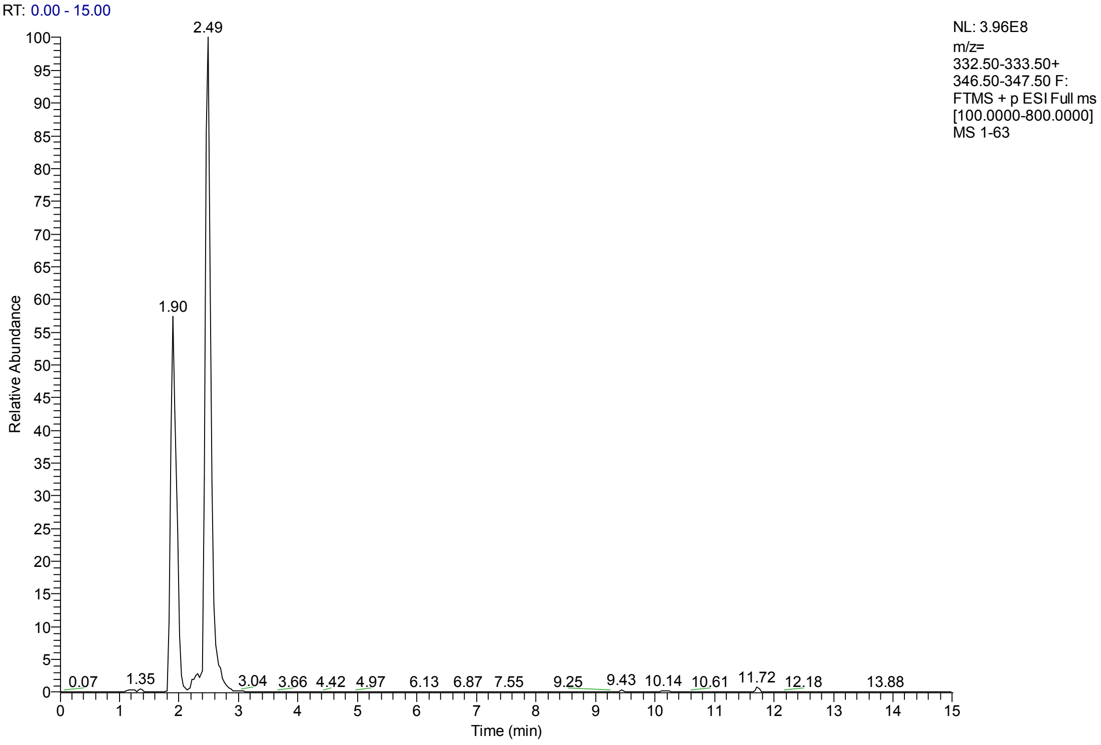

S17. Oscillatoria nigro-viridis PCC 7112(L-S)

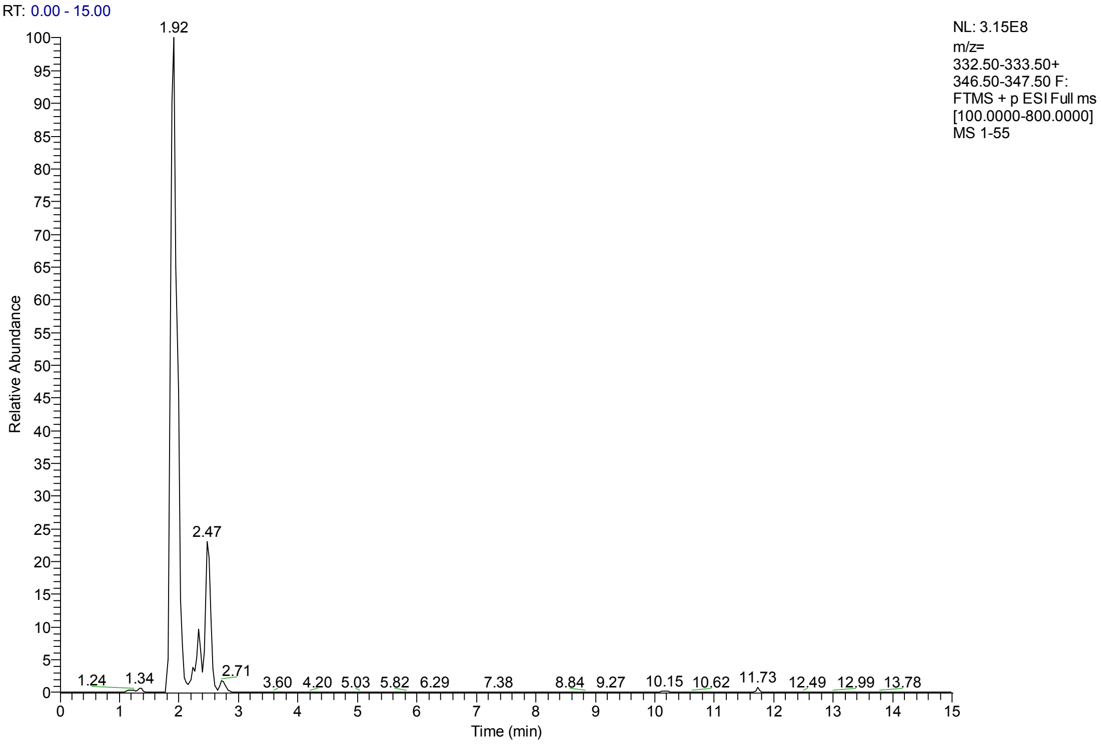

S18. Gloeocapsa sp. PCC 73106(F-A)

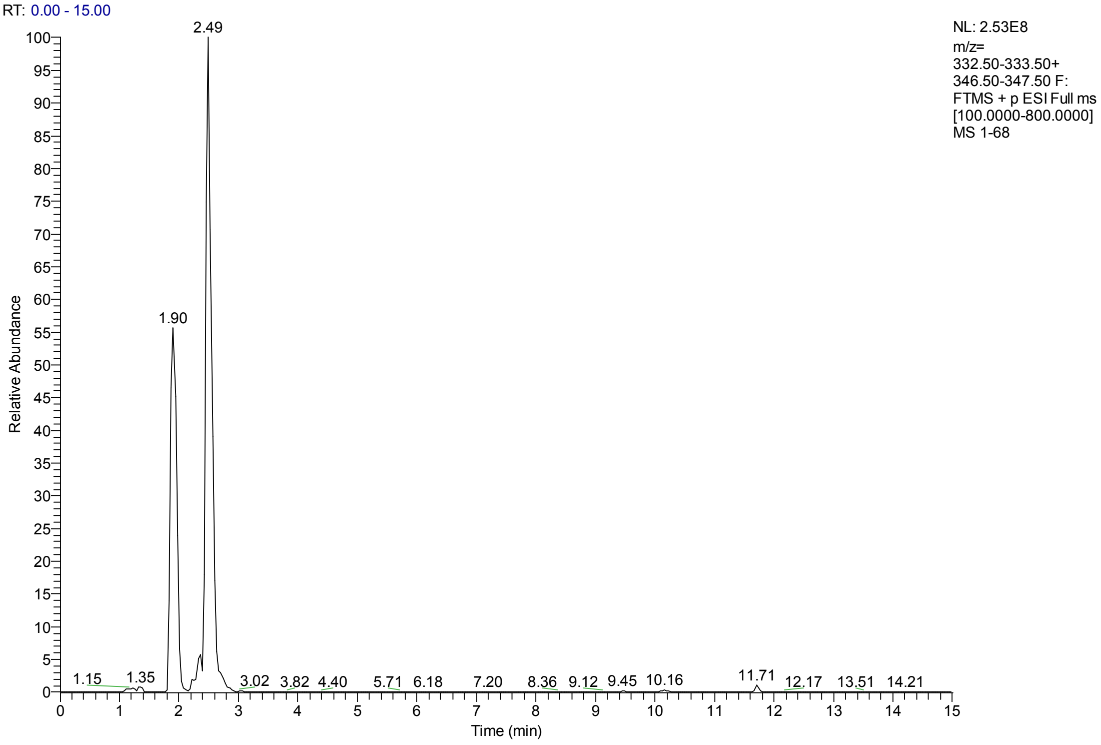

S19. Microcystis aeruginosa PCC 7806(C-A)

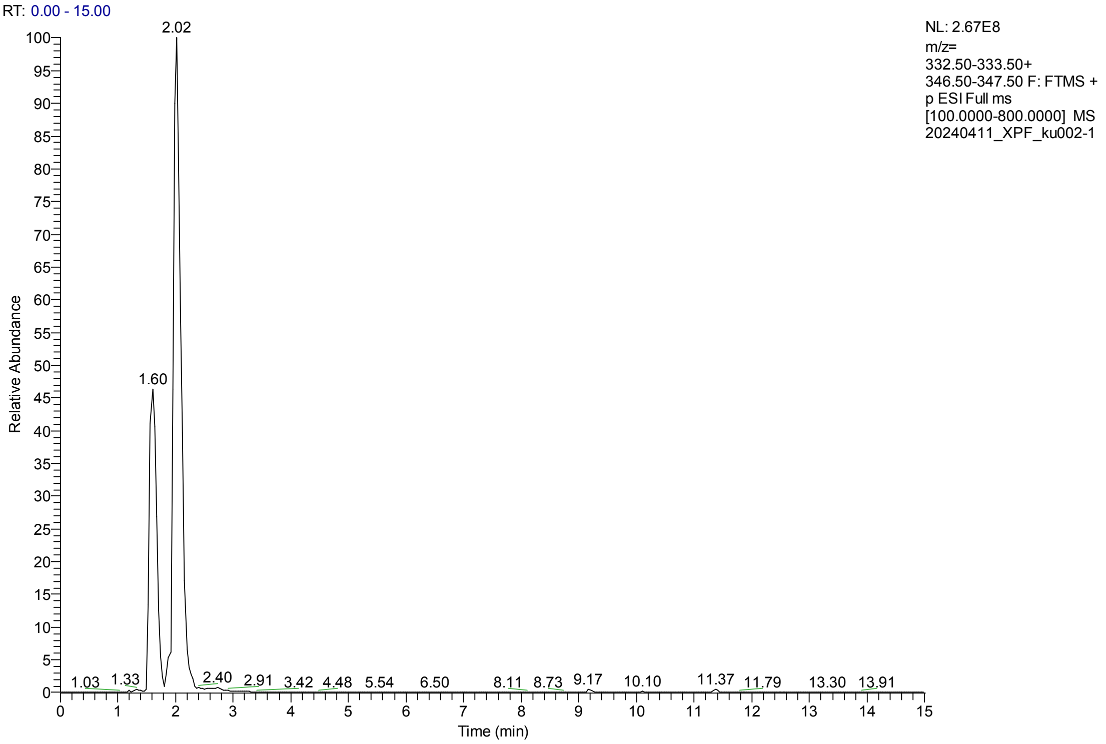

S20. Coleofasciculus chthonoplastes PCC 7420(C-A)

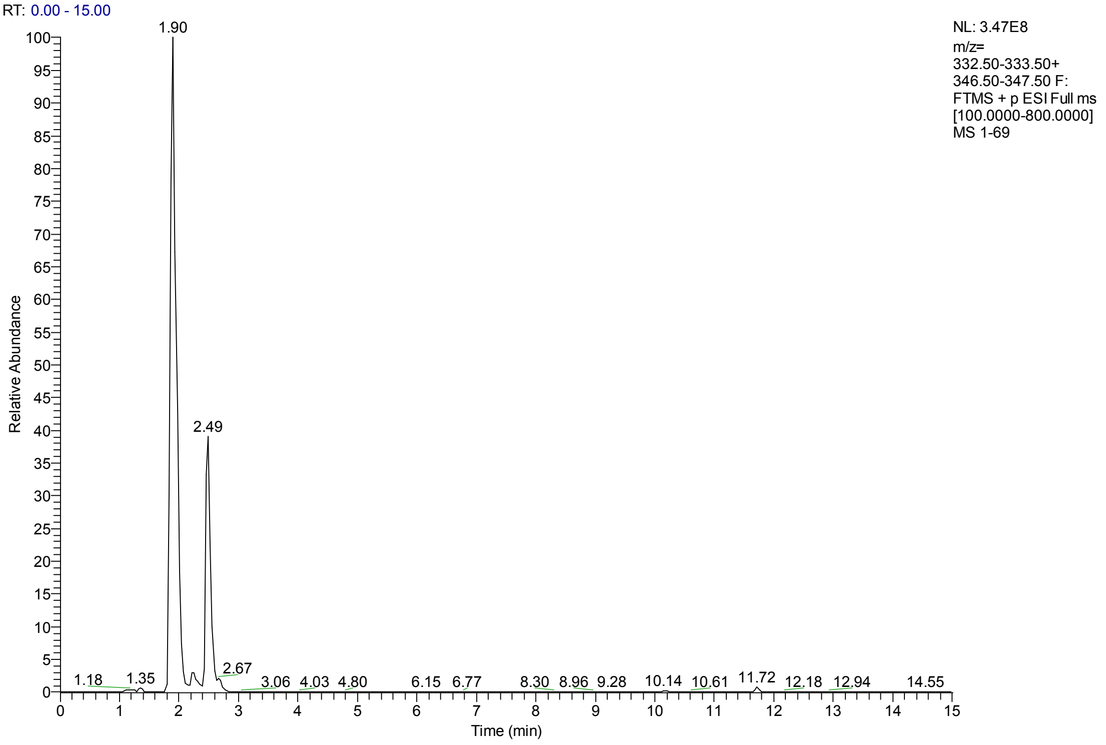

S21. Cyanothece sp. BG0011(Y-A)

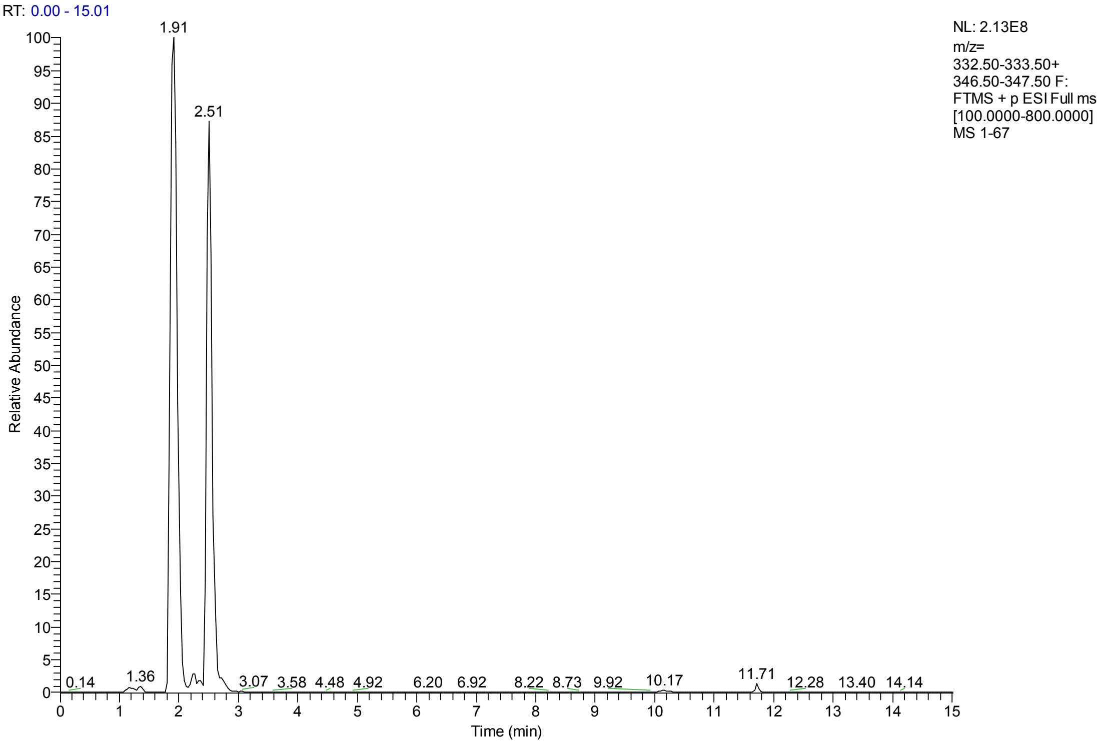

S22. Crocosphaera chwakensis CCY0110(Y-A)

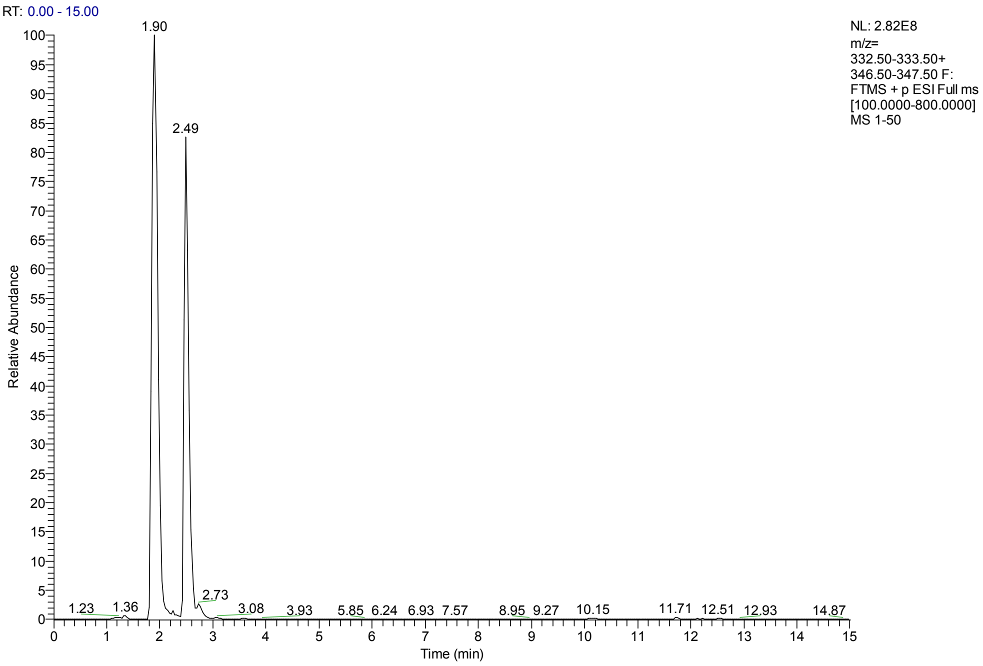

S23. Nostoc sp. PA-18-2419(Y-S)

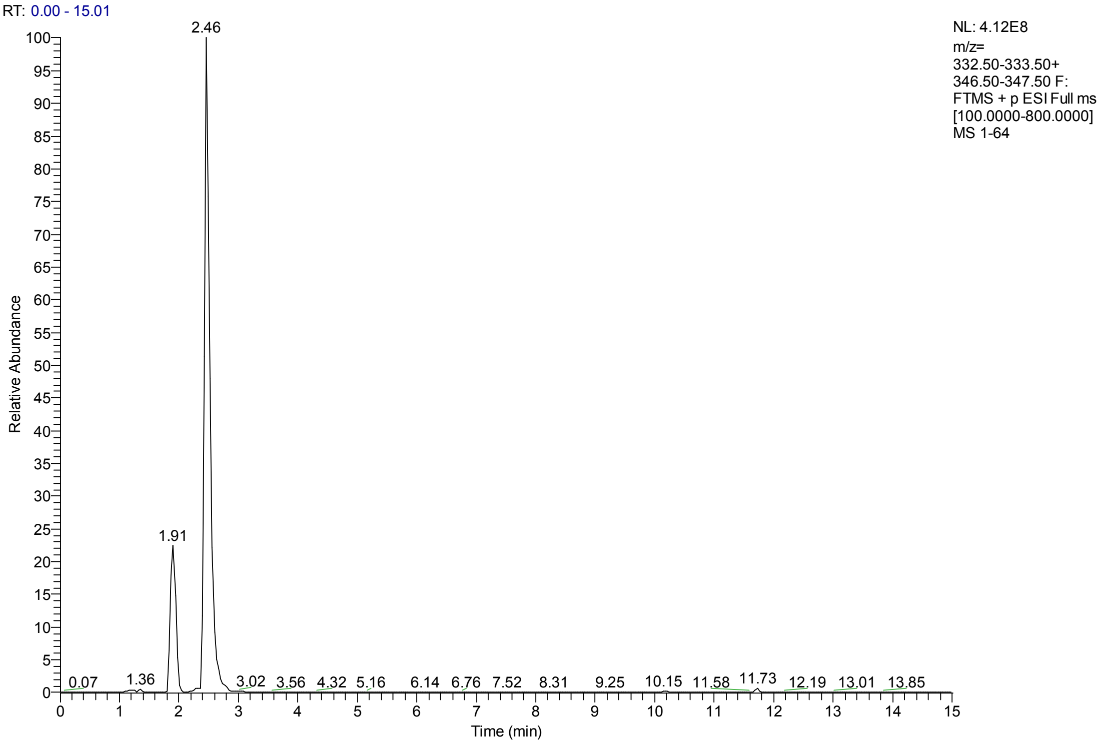

S24. Nostoc sp. NIES-4103(Y-S)

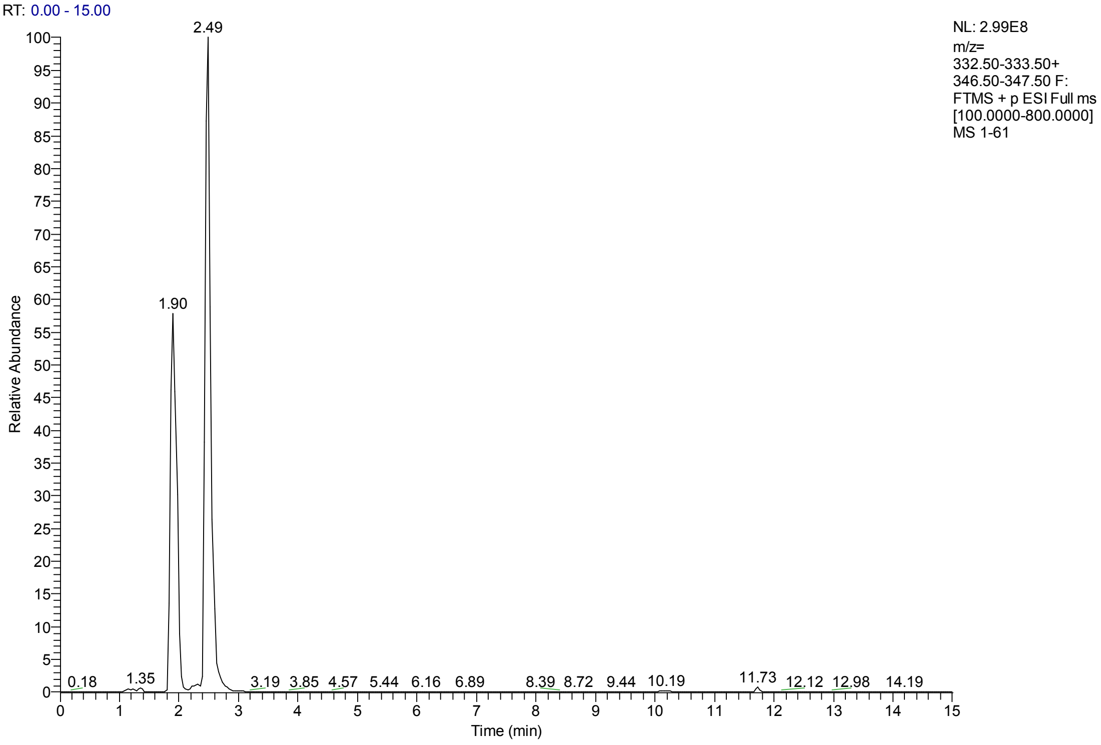

S25. Calothrix elsteri CCALA 953(A-S)

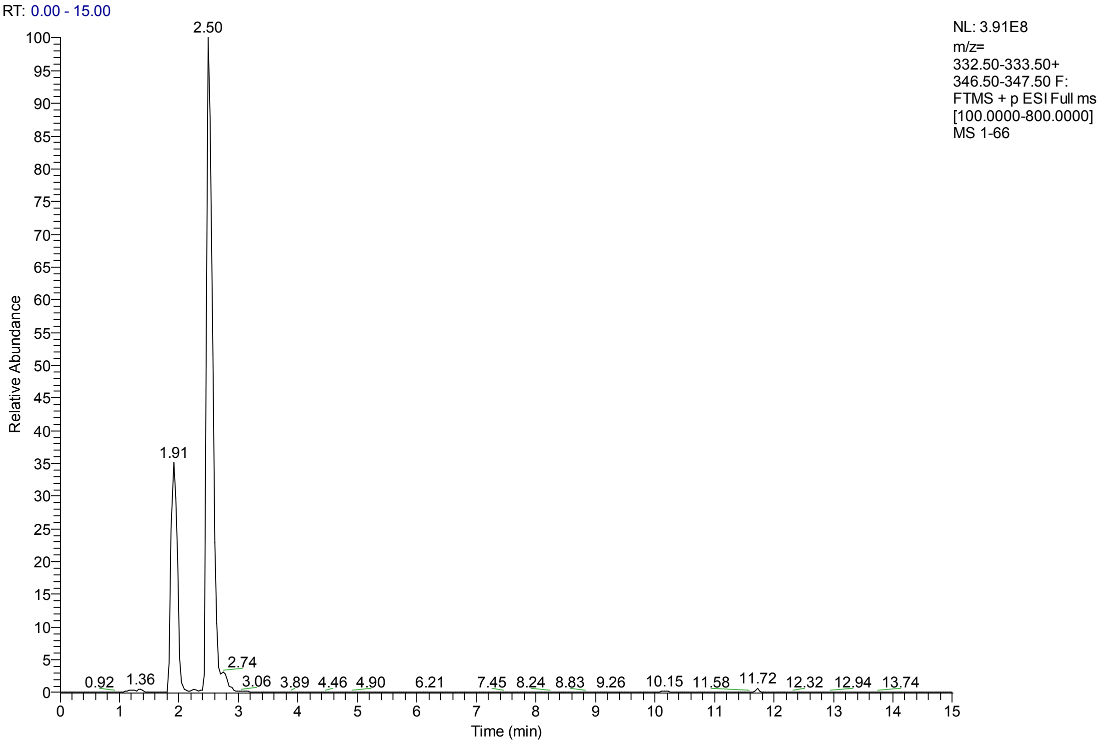

S26. Calothrix sp. PCC 6303(A-S)

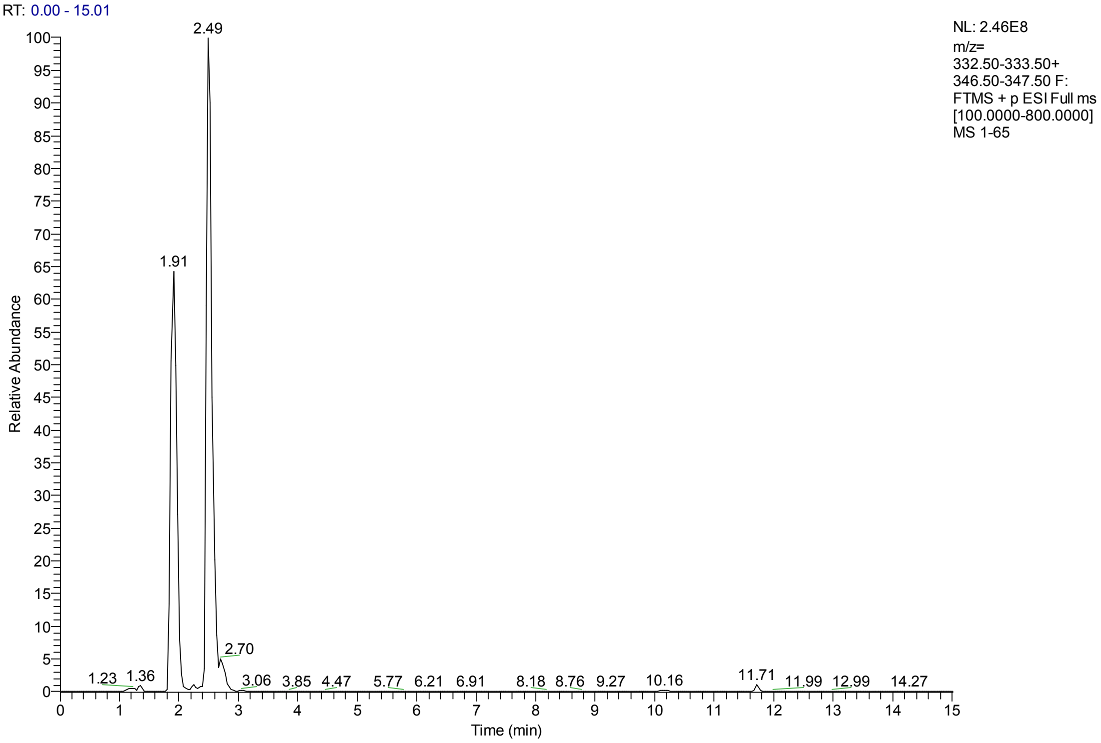

S27. Chamaesiphon polymorphus CCALA 037(M-S)

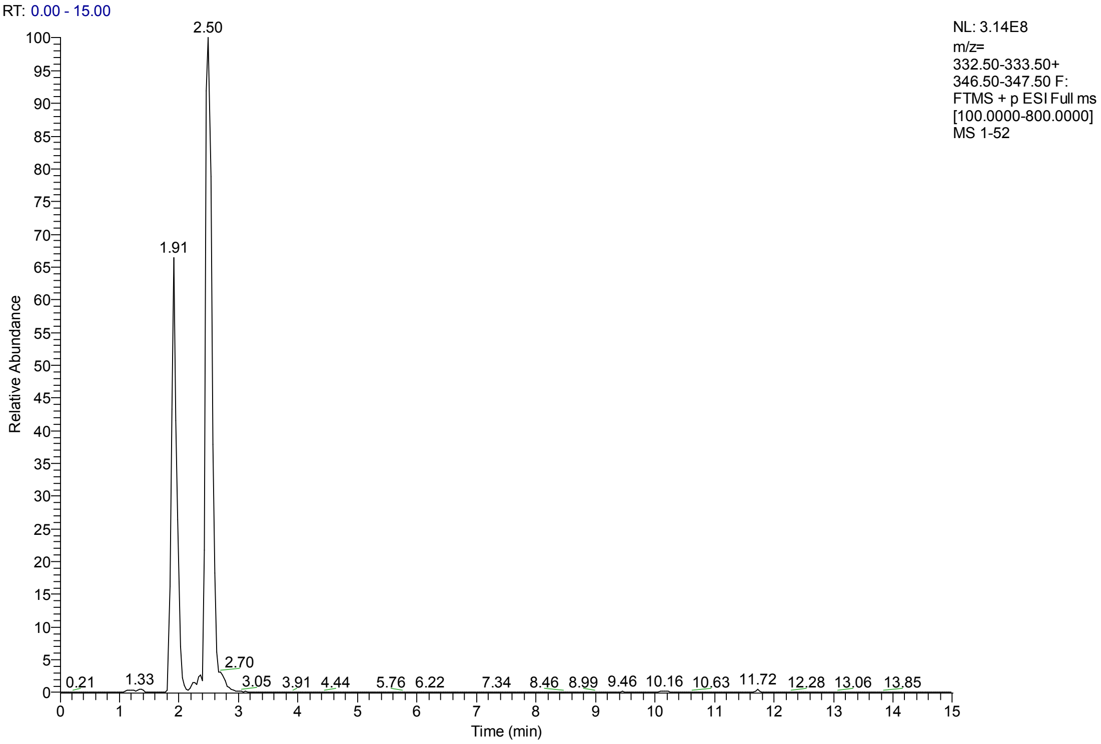

S28. Chamaesiphon minutus PCC 6605(M-S)

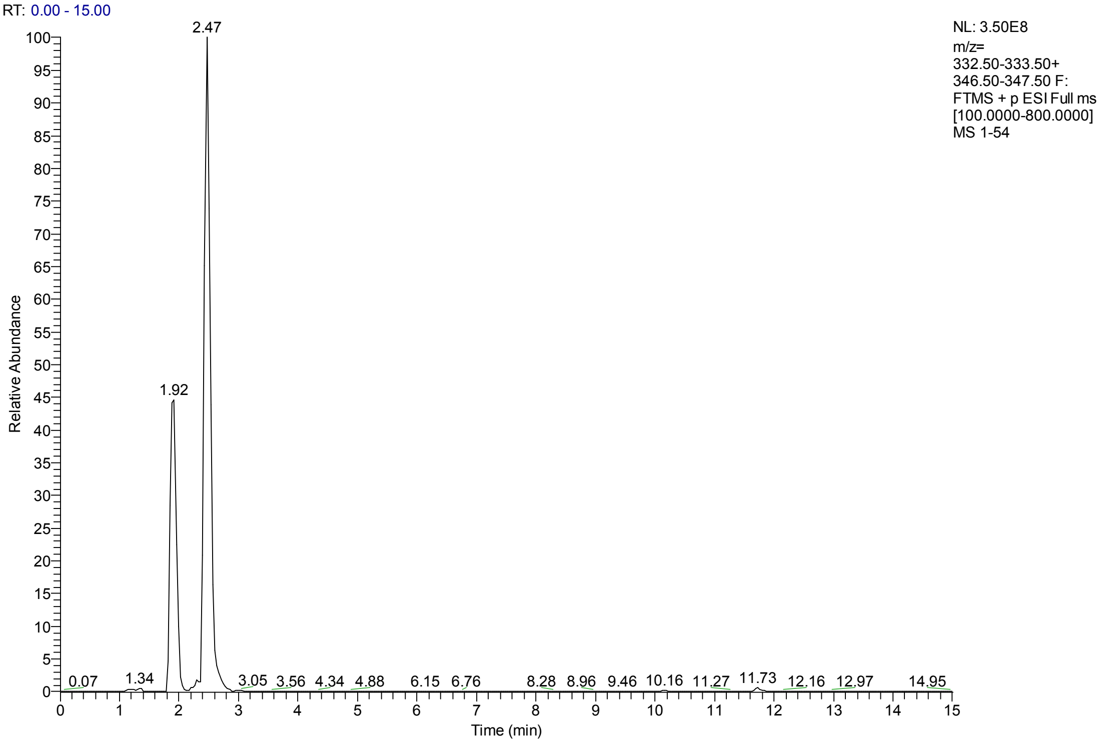

Supplement: Supplementary file 1 — ao5c01035_si_001.pdf [file ao5c01035_si_001.pdf]
